# *P/M* Macromolecular Switch Based on Conformational Control Exerted by an Achiral Side Chain within an Axially Chiral Locked Pendant

**DOI:** 10.1021/jacs.3c10766

**Published:** 2023-12-27

**Authors:** María Lago-Silva, María Magdalena Cid, Emilio Quiñoá, Félix Freire

**Affiliations:** † Centro Singular de Investigación en Química Biolóxica e Materiais Moleculares (CiQUS) and Departamento de Química Orgánica, Universidade de Santiago de Compostela, E-15782 Santiago de Compostela, Spain; ‡ Departamento de Química Orgánica, Campus Lagoas-Marcosende, Universidade de Vigo, E-36310 Vigo, Spain

## Abstract

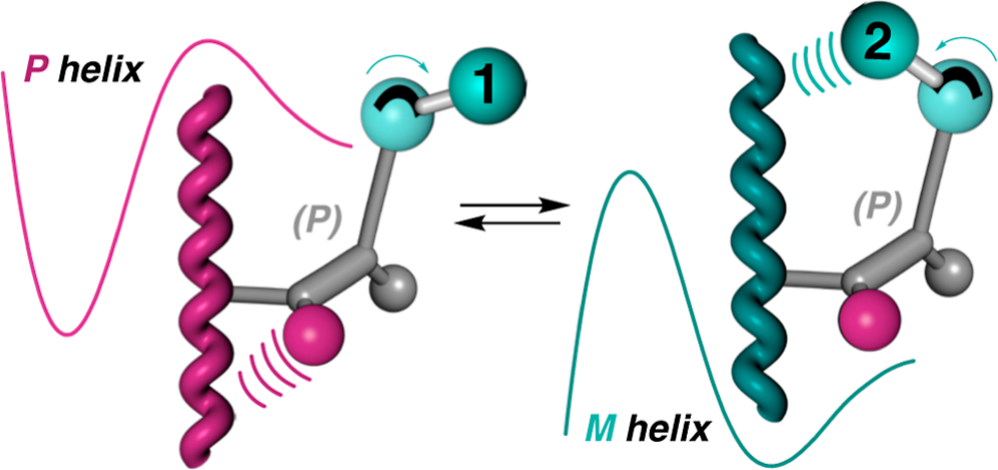

Molecular switches, supramolecular chemistry, and polymers can be combined to create stimuli-responsive multichiral materials. Therefore, by acting on the extended/bent conformational composition of an achiral arm, it is possible to create a macromolecular gear, where different supramolecular interactions can be activated/deactivated to control the helical sense of a polymer containing up to five different chiral axial motifs. For this, a chiral allene with a flexible achiral arm was introduced as a pendant in poly(phenylacetylene). Through flexible arm control between extended and bent conformations, it is possible to selectively induce either a *P* or *M* helical sense in the polymer, while the relative spatial distribution of the substituents in the allene remains unaltered in two perpendicular planes (configurationally locked). These results show that complex dynamic multichiral materials can be obtained by the polymerization of appropriate monomers that combine chirality, switching properties, and the ability to generate chiral supramolecular assemblies.

## Introduction

Molecular and macromolecular switches are mechanisms used by nature for signaling or transport processes. Thus, the interaction of biomolecular switches with different stimuli, such as pH, ions, and light, frequently led to an equilibrium between two functionally (ON/OFF) relevant conformational states.^1−3^ In general, elucidation of the mechanisms governing conformational control in large biomacromolecules is hampered by the complexity of the systems.^4^ To gain knowledge in this field, the scientific community has done exhaustive work over the last few decades to develop simple and easily tunable molecular or macromolecular switches,^5−13^ where the knowledge gained is of great use for subsequent designs. The molecular torsion balances of Wilcox et al.^14^ constitutes an interesting example. These systems inspired complex and sophisticated molecular machines such as the robotic arms developed by Leigh et al.^15^

Dynamic helical polymers are macromolecular switches^16−19^ where the *P/M* screw sense control is achieved by resorting to different helix induction mechanisms that arise from the conformational manipulation of the pendant of a monomer repeating unit because of interactions with different stimuli. Thus, information from the chiral center to the main chain of the polymer can be transmitted directly, across space, through helical induction effects such as tele-induction,^20−34^ chiral overpass,^35,36^ or substituent overpass,^37^ or indirectly, in a two-step process, where information from a chiral group placed at a remote position on the pendant is first transmitted to an achiral spacer and then harvested by the polyene backbone (chiral harvesting).^38−41^ In copolymers, Zentel et al. designed an isocyanate copolymer that shows reversible helix-inversion induced by isomerization of an azobenzene group.^18,19^

In this work, our objective is to create a macromolecular helical switch based on a chiral pendant that possesses, within its structure, a molecular machine formed by an achiral molecular torsion balance. More precisely, our goal is to control the *P* and *M* helical senses of a macromolecular gear without altering the chiral information on the pendant, and where conformational changes of an achiral side chain are the only requirement necessary to induce a helix inversion in the macromolecular polymer structure. Therefore, this novel helix induction mechanism will show how conformational changes in the achiral side chain of a chiral pendant result in helical sense control of the helix, without altering the chiral information of the pendant.

**Figure 1:**
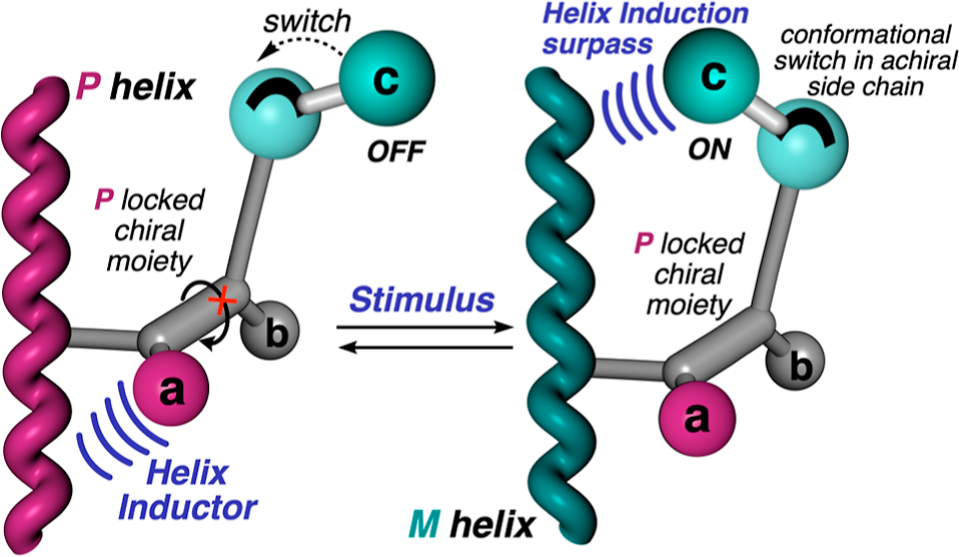
Conceptual view of a helix inversion process commanded by a mimetic molecular torsion balance as a pendant.

To achieve this goal, it is necessary to design a chiral and rigid pendant group that meets the following requirements (Figure 1): 1—the relative spatial distribution between the different substituents (**a**, **b**, **c**), with respect to the chiral element of the pendant, must be locked. For instance, the four substituents of a chiral allene are placed in two perpendicular planes, whose relative spatial distribution will always be the same due to the presence of two consecutive double bonds. 2—the bulkiness of the different substituents (**a**, **b**, **c**) must be different to establish a well-defined hierarchical helix induction effect among them. It is necessary to also consider the distance from the substituents to the polymer main chain to control their helix induction effects. 3—one of these substituents, i.e., **c** in Figure 1, must show different conformations when playing with intramolecular and/or intermolecular forces that function similarly as a molecular torsion balance.

Therefore, when this side chain adopts a conformation that places the bulky group remote from the backbone, the helix will be governed by a different substituent of the chiral pendant. However, by acting on the conformational composition of the molecular torsion balance mimetic side chain, this substituent will place its atoms close to the backbone, commanding a helix inversion of the polyene main chain. Therefore, our goal is to create a macromolecular gear where a conformational switch of an achiral side chain from a chiral pendant promotes a helix inversion of the polymer.

## Results and Discussion

**Figure 2:**
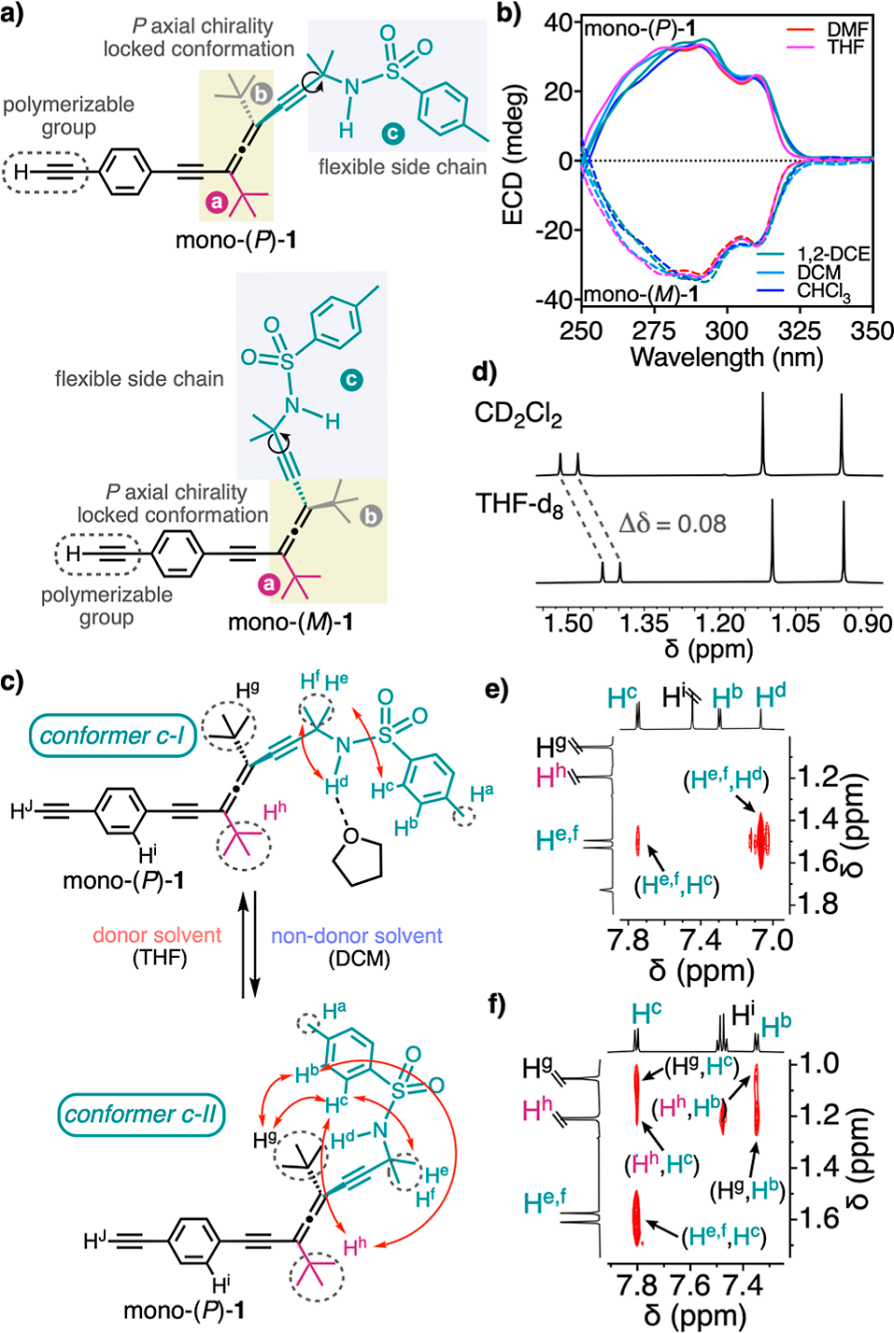
(a) Chemical structures of mono-(*P*)-**1** and mono-(*M*)-**1**. (b) ECD studies of mono-(*P*)-**1** and mono-(*M*)-**1** in different solvents (0.8 mM). (c) Conformational flexibility of the C—C—C—N bond (substituent **c**) (a–c labels are used to indicate substituents and do not refer to the priority of configuration) (red arrows indicate the most relevant protons showing the NOESY signal). (d) ^1^H NMR zoomed area of the two methyl (H^e,f^) and *tert*-butyl groups of mono-(*P*)-**1** in non-Lewis base (CD_2_Cl_2_) and Lewis base (THF-*d*_8_) solvents. (e) NOESY zoomed area (THF-*d*_8_, 278 K, 750 MHz). (f) NOESY zoomed-in area (CD_2_Cl_2_, 278 K, 750 MHz).

In a recent work, we demonstrated that poly[(allenylethynylenephenylene)acetylene]s (PAEPAs) are helical polymers whose screw sense excess is determined by the axial conformational stability of the chiral allene.^37^ Accordingly, PAEPAs comprise the two first requisites of the desired macromolecular gear, i.e., a unique spatial distribution of the different substituents in the two perpendicular planes of the allene. So, to complete our design, two *tert*-butyl groups were included in two perpendicular planes of the allene (side chains **a** and **b**) and a (dimethyl)methyl-*p*-tolyl-sulfonamide group in the last vacant position of the allene (side chain **c**), which can easily form hydrogen bonds with Lewis base solvents or anions^42^ (Figure 2a). Therefore, monomers mono-(*P*)-**1** and mono-(*M*)-**1** were synthetized from previously reported allenes^37,43,44^ by derivatizing the propargylic alcohol with a sulfonamide group using FeCl_3_ as a catalyst (Section 2 of the Supporting Information). ECD studies of both monomeric enantiomers in different solvents show, as expected, that the *P* or *M* axial chirality of the allene moiety remains unaltered due to restricted rotation along the two consecutive double bonds of the allene (Figure 2b). However, the substituent bearing the sulfonamide group of mono-(*P*)-**1** can adopt two different conformations—c-I and c-II (Figure 2c)—in the Lewis base (c-I: THF, DMF, and DMSO) and non-Lewis base solvents (c-II: CHCl_3_, DCM, and 1,2-DCE) as inferred from ^1^H NMR studies. In them, the protons of the two methyl groups (H^e,f^) of the carbon linked to the sulfonamide group shift upfield when mono-(*P*)-**1** dissolves in Lewis base solvents (Figures 2d and S12) due to the anisotropic effect of the *p*-tolyl group. This conformational change is produced by a H-bond interaction between the Lewis base solvents and the acidic proton (H^d^) of the sulfonamide. Furthermore, to elucidate the most stable conformers adopted by mono-(*P*)-**1** in Lewis base and non-Lewis base solvents, NOESY experiments were carried out (Figures 2e,f, S13b,c, and S14b). From these studies, it was possible to obtain the distance from the sulfonamide proton (H^d^) to the two methyl groups of the carbon linked to the sulfonamide group, in addition to the distance from these two methyl groups to the *p*-tolyl group (Table S3). Thus, while in Lewis base solvents, the *p*-tolyl-sulfonamide group adopts an extended arrangement (conformer c-I), in non-Lewis base solvents, a bent conformation is generated placing the *p*-tolyl-sulfonamide group closer to the alkyne (conformer c-II) (Figure 2c).

**Figure 3:**
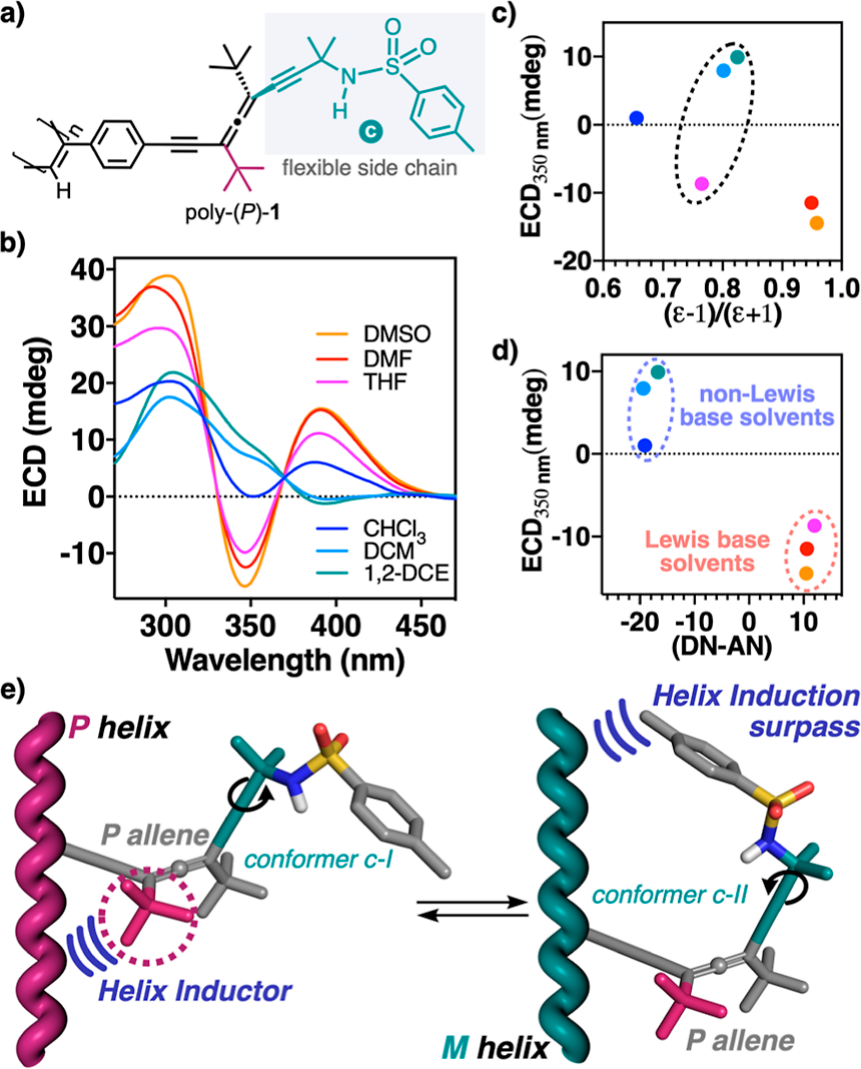
(a) Chemical structure of poly-(*P*)-**1**. (b) ECD studies of poly-(*P*)-**1** in different solvents (0.8 mM). (c) Graph showing the relationship between the ECD signal at 350 nm and the polarity of the solvent. (d) Graph indicating the relationship between the Lewis base/acceptor properties of the solvents and the ECD signal at 350 nm. (e) Conceptual representation of the effect of a conformational change at the pendants by external stimuli.

Next, monomers (*P*)-**1** and (*M*)-**1** were polymerized using [Rh(nbd)Cl]_2_ as the catalyst,^45,46^ affording poly-(*P*)-**1** and poly-(*M*)-**1** (Figure 3a) in good yields (85%), low polydispersity and with high contents of cis configuration of the double bonds as inferred from ^1^H NMR and Raman studies (Figures S6 and S8).^47,48^

ECD and UV–vis studies were carried out for poly-(*P*)-**1** and poly-(*M*)-**1** dissolved in solvents with different polarities and Lewis base character (Figures 3b and S15–S17), which allowed us to determine their stimuli-responsive properties by controlling the conformational composition of the side chain containing the sulfonamide group (side chain **c**) (Figure 3a). So, ECD studies in Lewis base solvents such as DMF, DMSO, or THF, where the N–H of the sulfonamide group can establish a hydrogen bond interaction with the solvent, show that the polymers adopt a *P* helix for poly-(*P*)-**1** and an *M* for poly-(*M*)-**1** with the classical three alternating Cotton bands [poly-(P)-**1** ECD(+/-/+); poly-(M)-**1** ECD(-/+/-)] (Figures 3b, S15, and S16). On the other hand, when the polymers are dissolved in non-Lewis base solvents such as DCM or 1,2-DCE, structural changes are observed in the polymers toward the opposite helical sense structures. Thus, poly-(*P*)-**1** shows a first ECD band slightly negative (ECD_350_ < 0; *M* helix), while the other two are positive (-/+/+) (Figure 3b), whereas for poly-(*M*)-**1**, the first ECD band becomes slightly positive (ECD_350_ > 0; *P* helix), and the other two are negative (+/-/−) (Figure S16). To demonstrate that these structural changes are attributed to the Lewis base character of the solvents and not to their polarity, the ECD response of poly-(*P*)-**1** was plotted against polarity and Lewis base/acceptor properties^30,49^ of the solvents quantitatively represented by their dielectric constant (ε) and Gutmann’s values (Figure 3c,d). Thus, by plotting the ECD signal at 350 nm versus the solvent dielectric constant (ε), it is possible to observe how THF, with a polarity like DCM or DCE, produces an ECD spectrum with opposite sign (Figure 3c). On the other hand, when the ECD changes at 350 nm are plotted versus the solvents Gutmann’s values (DN-AN) (Figure 3d), it is possible to visualize how Lewis base solvents promote a negative Cotton band in this region (ECD_350_ < 0), whereas non-Lewis base solvents promote a positive Cotton band (ECD_350_ > 0). These studies show the structural changes produced in poly-(*P*)-**1** are due to the different Lewis base character of the solvents that cause the conformational changes in the allenic sulfonamide (chain **c**). An analogous behavior was observed for poly-(*M*)-**1** (Figure S16a). This interaction was also inferred by IR studies where the frequency of the NH of the sulfonamide group of poly-(*P*)-**1** shows a Δν = 31 cm^–1^ in THF (3276 cm^–1^) with respect to the value obtained in 1,2-DCE (3245 cm^–1^) due to its supramolecular interaction with the Lewis base solvent (Figure S19).

To further corroborate these results, STD NMR experiments were carried out for solutions of poly-(*P*)-**1** in THF-*d*_8_ and CD_2_Cl_2_, which revealed a poly-(*P*)-**1**/THF-*d*_8_ supramolecular interaction (Figure S20), while no effects were observed in CD_2_Cl_2_ (Figure S21). Moreover, NOESY NMR experiments for poly-(*P*)-**1** demonstrated that the *p*-tolyl-sulfonamide group adopts an extended conformation and therefore lies away from the polyene backbone (conformer c-I) in Lewis base solvents, whereas in non-Lewis base solvents, a bent conformation is favored (conformer c-II) (Figures 3e and S22 and S23).

UV–vis studies indicate that the diastereomeric *P* and *M* macromolecular helices obtained in Lewis base and non-Lewis base solvents at room temperature have similar scaffolds because no variations are observed in the conjugation of alternating double bonds (band at ca. 380 nm) due to stretching or compression of the polyene main chain (Figure S17b).

**Figure 4:**
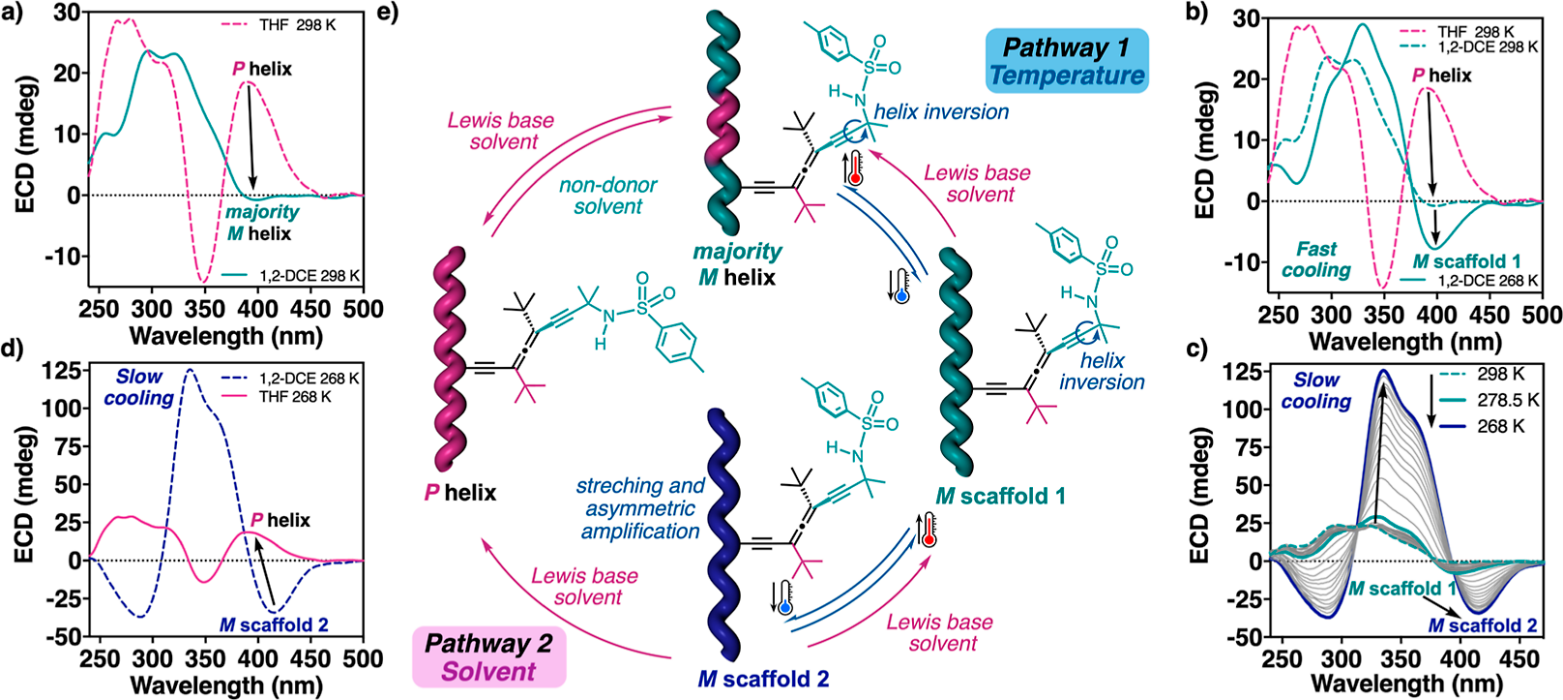
(a) Evolution of the ECD spectra of poly-(*P*)-**1** (1.6 mM) from THF to 1,2-DCE solutions at rt. (b) VT-ECD spectra of poly-(*P*)-**1** (1.6 mM in 1,2-DCE) after fast cooling. (c) VT-ECD spectra of poly-(P)-**1** (1.6 mM in 1,2-DCE) after slow cooling. (c) VT-ECD spectra of poly-(*P*)-**1** in 1,2-DCE (1.6 mM) after slow cooling followed by the addition of 60 equiv of THF at 268 K. (e) Schematic representation of reversible tuning of the backbone scaffold under external stimuli.

This macromolecular chirality switch (*P* to *M* or *M* to *P*) can be attributed to only the side chain of the chiral allene (Figure 3a) because it is the only flexible moiety in the pendant group. Thus, the conformational composition found in substituent **c** in the monomer also works in polymers (Figure 2c). These two conformers generate different steric effects when placed within a PAEPA helical scaffold: while conformer c-I fits well into this helix due to an extended orientation of the pendant, conformer c-II exhibits more steric interactions due to its bent conformation, so poly-(*P*)-**1** is better folded in Lewis base than in non-Lewis base solvents (Figure 4a).

To stabilize, on the pendant group of poly-(*P*)-**1**, conformer c-I in Lewis base solvents and conformer c-II in non-Lewis base solvents, low-temperature ECD studies were carried out at 1,2-DCE and THF. When Lewis base solvents are used (e.g., THF), no helical changes are observed at either high or low temperatures or at different heating/cooling rates, indicating a quasi-static thermal behavior of the polymer (Figures S24a and S25a). In this case, the rigidity of the allene pendant in combination with the adoption of a very stable extended conformer of side chain **c** results in a well folded helix (Figure 4e). Conversely, when VT-ECD studies were carried out for poly-(*P*)-**1** in 1,2-DCE, a different thermal effect was observed depending on the cooling rate. Thus, when a cuvette containing the poly-(P)-**1** solution was placed directly in a cooled bath at 268 K, a magnification of the ECD trace was observed (Figures 4b and S24c), indicating an enhancement of the *M* screw sense preference in the polymer due to stabilization of conformer c-II of side chain **c** at the pendant (*M* scaffold 1).

Intriguingly, when the temperature of the 1,2-DCE solution was lowered by using a cooling rate (≤10 K/min), a two-step helix induction process was obtained. In an initial stage, when the temperature was lowered to 278.5 K, a screw sense excess of the *M* helix adopted by poly-(*P*)-**1** was obtained, like when applying a fast cooling (Figures 4b and S24c). However, when the temperature decreases from 278.5 to 268.0 K, a second stretched *M* helix (*M* scaffold 2) is formed (Figures 4c, S25c,d, and S26). This structural change is fully reversible, and when the temperature increases from 268 to 278 K, the *M* scaffold 1 is recovered, indicating that to obtain the *M* scaffold 2 it is necessary to go through the *M* scaffold 1 (Figures 4e and S27). Interestingly, by looking at the variation of the ECD trace during the cooling process (Figure 4c), it is possible to observe that the stretching of the polyene band (maximum absorbance of the ECD polyene band: 400 nm for *M* scaffold 1 and 420 nm for *M* scaffold 2) is accompanied by the emergence of an abnormally high positive exciton coupling band centered at 337 nm, which corresponds to the absorbance of the chiral allene pendant. Analogous results were also obtained for the enantiomer poly-(*M*)-**1** (Figure S29).

To further demonstrate that conformational changes around the sulfonamide group are responsible for the helix inversion, monomer (*P*)-**1** was methylated at the sulfonamide group [(*P*)-**2**] (see the Supporting Information). As expected, poly-(*P*)-**2** adopts, in all solvents at 293 K, a *P* screw sense excess commanded by the *P* chirality of the allene (Figure S18), which increases when the ECD spectra are recorded at lower temperatures (Figures S30–S33).

Recently, our group reported that the use of rigid and planar spacers such as oligophenylenethynylenes (OPEs) or bispyridyldichlorido Pt(II) complexes between the polyene backbone and the chiral pendant^40,41^ can produce, in certain scaffolds, a new chiral axial arrangement of the spacers within the helical scaffold whose ECD pattern dominates the ECD spectra of the whole helical system. In these polymers, helix induction in the polyene backbone follows a chiral harvesting mechanism, where chiral information from the axial array of the spacer is harvested by the polyene backbone.

In this sense, when the 3D-structure of mono-(*P*)-**1**, an extended and rigid aryl-alkyne-allene fragment is found that could produce a novel axial chiral motif within the helical scaffold. To discern 3D structural parameters of poly-(*P*)-**1** in Lewis base and non-Lewis base solvents, atomic force microscopy (AFM) studies were performed. Thus, 2D crystals of poly-(*P*)-**1** were prepared from THF and 1,2-DCE solutions following Yashima’s protocol^50^ and employing highly oriented pyrolytic graphite (HOPG) as the substrate. High-resolution images revealed the presence of well-ordered monolayers in both cases: external *M* helix (THF solution) and external *P* helix (1,2-DCE solution).

**Figure 5:**
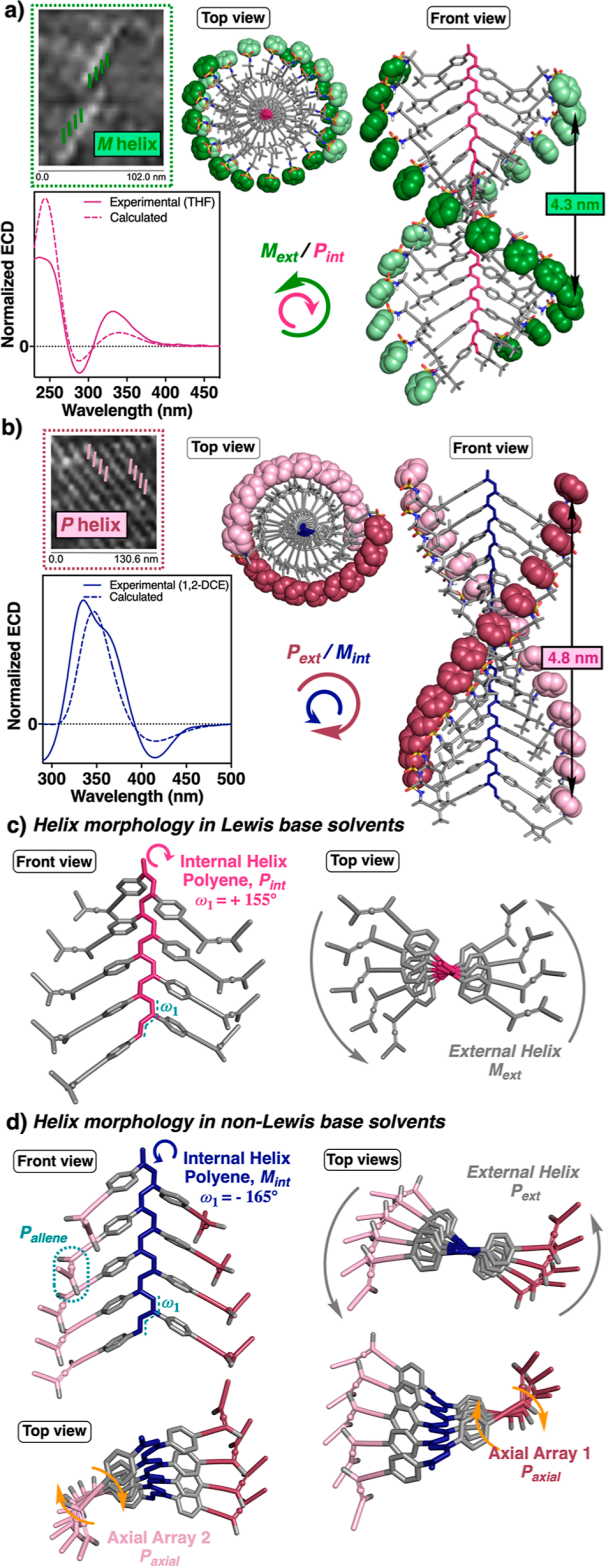
3D model structures of poly-(*P*)-**1** obtained from the AFM image of 2D crystals in (a) Lewis base and (b) non-Lewis base solvents. ECD spectra of poly-(*P*)-**1** in (a) THF and (b) 1,2-DCE vs calculated from the 3D structures obtained in both solvents. Axial motifs found in the helical structures adopted by poly-(*P*)-**1** in (c) Lewis base and (d) non-Lewis base solvents.

In addition, other important structural parameters were obtained, such as a helical pitch of 4.3 nm for the THF sample and a longer pitch (4.8 nm) for the 1,2-DCE sample (Figures 5a,b and S34 and S35). DSC studies corroborated the presence of a cis–transoidal polyene skeleton (Figure S9). So, the combination of all the obtained data allowed us to model an approximated 3D structure for poly-(*P*)-**1** in Lewis base (*P* internal helix, ω_1_ = 155°) and non-Lewis base solvents (*M* internal helix, ω_1_ = −165°) (Figure 5 and Section 15 of the Supporting Information). Consequently, in the helical structure with ω_1_ = 155°, the two classical coaxial helices are found to be—internal polyene; external pendants (Figure 5c). However, in the most stretched helix (ω_1_ = 165°), a new axial motif was disclosed by ECD. It is originated by a supramolecular chiral array of the allene groups (Figure 5d), forming a helix that rotates in the opposite direction from the polyene backbone, as shown by the negative Cotton effect at 420 nm. Furthermore, a positive exciton coupling band centered at 337 nm is indicative of a positive tilting degree in the axial array of the allene groups (Figure S38).

Thus, the allene group not only functions as a helix inductor due to its intrinsic chirality, but creates a supramolecular array within the helical scaffold of the polymer, generating yet another chiral motif, previously observed only for nonchiral planar spacers as OPEs or bispyridyldichlorido Pt(II) complexes.^40,41^ Consequently, five different axial motifs are found in a PAEPA: the allene, the internal helix, the external helix and two allene axial arrays (*P*_allene_, *M*_int_/*P*_ext_/*P*_axial-1_/*P*_axial-2_) (Figure 5d).

**Figure 6:**
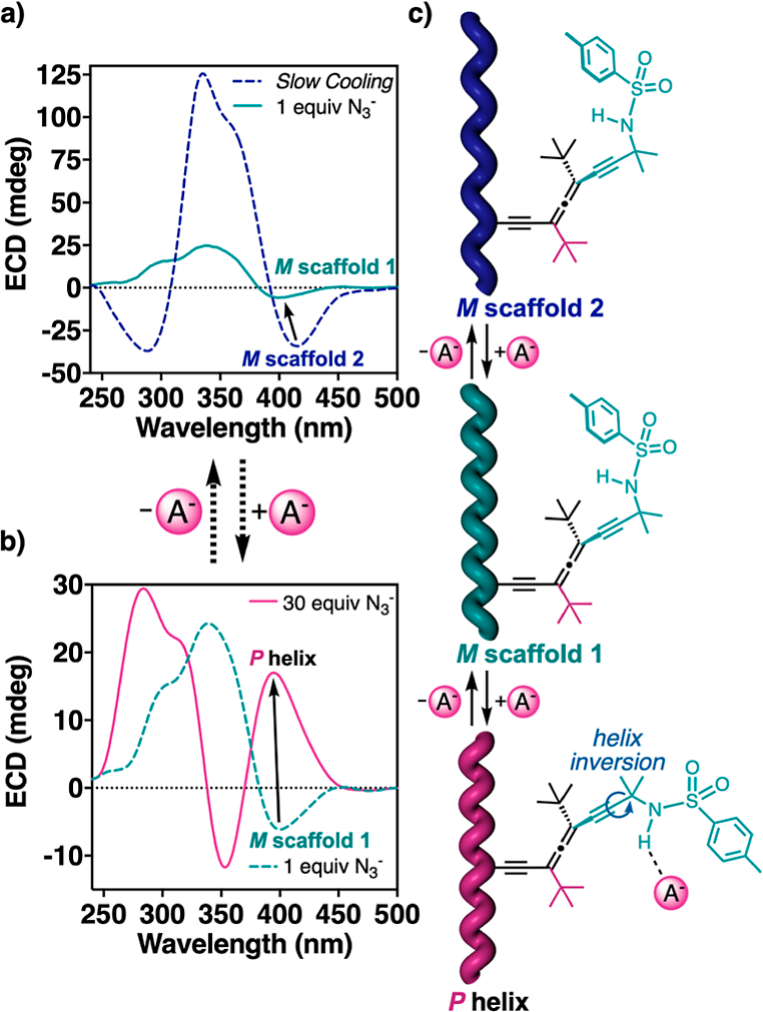
(a) ECD spectra of poly-(*P*)-**1** (1.6 mM in 1,2-DCE, 268 K) after slow cooling (dashed blue) and (b) after being titrated with the azide ion [1 equiv (green) and 30 equiv (pink)]. (c) Schematic representation of the reversible switching of the helical sense of poly-(*P*)-**1** upon the addition of the azide ion.

Finally, to further demonstrate that the dynamic behavior of poly-(*P*)-**1** is due to variations in the conformational composition of side chain **c** containing the sulfonamide group, a titration with different anions such as N_3_^–^, CN^–^, and F^–^ (introduced as tetrabutylammonium salts: TBAN_3_, TBACN, TBAF, 0.35 mM in MeCN) were added to 1,2-DCE solutions of poly-(*P*)-**1** slowly cooled at 268 K whose side chain **c** adopted, consequently, a bent conformer (Figure 6 for TBAN_3_ and Sections 16 and 17 of the Supporting Information for other salts).

Thus, in the presence of 1 equiv of azide (Figure 6a), the stretched *M* helix (*M* scaffold 2) is transformed into a more compressed *M* helix (*M* scaffold 1), where an equilibrium between bent and extended structures at the side chain of the allene is present. The reversibility of the process was demonstrated by extraction with water of the anion salt (TBAN_3_) (see Figure S41). By increasing the amount of the azide anion (>3 equiv), a helix inversion arises because of the adoption of an extended structure in substituent **c** (Figures 6b and S40a). Therefore, these results reveal the role of the sulfonamide group and the extended/bent orientation of substituent **c** in the helix inversion of poly-(*P*)-**1** (Figure 6c).

## Conclusions

In conclusion, we have demonstrated that the helical sense of a chiral helical polymer, such as a PAEPA, can be modulated by acting on the conformational composition of an achiral side chain without changing the relative spatial distribution of the substituents in an axial chiral allene group used as pendant. So, the chiral allene will always have the same relative orientation of its substituents, showing a quasi-static behavior due to its conformational stability. In this work, we managed to create a dynamic PAEPA by altering the priority of the helix induction order due to the conformational control of an achiral side chain that works as a flexible arm. Thus, when this arm is extended, this substituent is placed away from the polyene backbone and does not interfere with the helix induction command ordered by the closer allene substituent. However, when the arm is bent, this substituent surpasses the order given by that substituent as it approaches the backbone and thus commands the helical sense of the PPA. As a result, a helix inversion mechanism of a dynamic helical polymer based on the conformational control of a flexible arm is presented. Moreover, from these studies we also found that multichiral helical structures, which comprise five axial motifs—the two coaxial helices (internal and external), the chiral allene, and two extra chiral axial motifs described by the aryl–ethynyl–allene array—can be prepared by combining rigid, extended, and axially chiral allenes with helical polymers such as poly(phenylacetylene)s. These results indicate that the combination of information or ideas from molecular switches, supramolecular chemistry, and polymers can give rise to different helical induction mechanisms through complex structures, allowing a better understanding of how information can be transferred at different levels of complexity.

## Supporting Information Available

Materials and methods; synthesis of monomers; synthesis of polymers; GPC studies; NMR Raman experiments; thermal studies; ECD studies of mono-(M)-**1**; Lewis and non-Lewis solvent effects in the conformational composition of mono-(P)-**1**; ECD studies of polymers; ATR/FT-IR studies; Lewis and non-Lewis solvent effects in the conformational composition of poly-(P)-**1**; low-temperature ECD studies; VT-ECD experiments; AFM images; theoretical calculations; and ECD, UV–vis, and NMR spectra of anion titration studies (PDF)

## Abbreviations

*P*: plus
*M*: minus
PAEPA: poly[(allenylethynylenephenylene)acetylene]s
ECD: electronic circular dichroism
THF: tetrahydrofuran
DMF: *N*,*N*-dimethylformamide
DMSO: dimethyl sulfoxide
DCM: dichloromethane
1,2,-DCE: 1,2-dichloroethane
NMR: nuclear magnetic resonance
NOESY: nuclear overhauser effect spectroscopy
UV–vis: ultraviolet–visible
IR: infrared spectroscopy
STD: saturation-transfer difference
VT: variable temperature
OPE: oligophenylenethynylene
AFM: atomic force microscopy
HOPG: highly oriented pyrolytic graphite
TBAX: tetrabutylammonium salt
