# Supplementary material for: P/M Macromolecular Switch Based on Conformational Control Exerted by an Achiral Side Chain within an Axially Chiral Locked Pendant
Source: J Am Chem Soc. 2023 Dec 27;146(1):752–9. doi: 10.1021/jacs.3c10766 (PMC10786024; doi:10.1021/jacs.3c10766)
Supplement: Supplementary file 1 — ja3c10766_si_001.pdf [file ja3c10766_si_001.pdf]

P/M Macromolecular Switch Based on Conformational Control exerted by Achiral Side Chain Within an Axially Chiral Locked Pendant

María Lago-Silva,[a] María Magdalena Cid,[b] Emilio Quiñoá[a] and Félix Freire[a]*

[a]*Centro Singular de Investigación en Química Biolóxica e Materiais Moleculares (CiQUS) and Departamento de Química Orgánica. Univer-sidade de Santiago de Compostela, E-15782 Santiago de Compostela, Spain.*
[b]*Departamento de Química Orgánica. Campus Lagoas-Marcosende, Universidade de Vigo, Vigo, E-36310, Spain.*

felix.freire@usc.es

http://felixfreire.com

Supporting Information

Contents

1. Materials and Methods **4**

2. Synthesis of Monomers **6**

Di-*tert*-butyl-2-methyl-9-(4-((trimethylsilyl)ethynyl)phenyl)nona-5,6-dien-3,8-diyn-2-ol (**S1**) 6

(*P*)-*N*-(5,7-di-*tert*-butyl-2-methyl-9-(4-((trimethylsilyl)ethynyl)phenyl)nona-5,6-dien-3,8-diyn-2-yl)-4-methylbenzenesulfonamide (**S2**) 8

(*P)*-Di-*tert*-butyl-9-(4-ethynylphenyl)-2-methylnona-5,6-dien-3,8-diyn-2-ol (mono-(*P*)-**1**) 10

(*M*)-Di-*tert*-butyl-9-(4-ethynylphenyl)-2-methylnona-5,6-dien-3,8-diyn-2-ol (mono-(*M*)-**1**) 12

(*M*)-*N*-(5,7-di-*tert*-butyl-9-(4-ethynylphenyl)-2-methylnona-5,6-dien-3,8-diyn-2-yl)-*N*,4-dimethylbenzenesulfonamide (mono-(*P*)-**2**) 13

3. Synthesis of polymers **15**

General procedure for polymerization 15

4. GPC Studies **17**

5. Raman Experiments **17**

6. Thermal Studies **18**

DSC Studies 18

TGA studies 18

7. ECD Studies of mono-(*M*)-1 .... **19**

8. Studies of the effect of Lewis base and non-Lewis base solvents on the conformation of mono-(*P*)-1 by NMR **19**

^{1}H-NMR studies of mono-(*P*)-**1** in non-Lewis base and Lewis base solvents 19

NOESY-NMR studies of mono-(*P*)-**1** in Lewis base and non-Lewis base solvents 20

9. ECD Studies of polymers **22**

ECD Studies of poly-(*P*)-**1** 22

ECD Studies of poly-(*M*)-**1** 22

Comparison of (*P*)- and (*M*)-enantiomers 23

ECD Studies of poly-(*P*)**-2** 23

10. ATR/FT-IR Studies **23**

11. Studies of the effect of Lewis base and non-Lewis base solvents on the conformation of poly-(*P*)-1 by NMR **24**

STD-NMR studies of the poly-(*P*)-**1** in Lewis base solvents and non-Lewis base solvents 24

NOESY-NMR studies of the poly-(*P*)-**1** in Lewis base and non-Lewis base solvents 27

12. Low Temperature ECD Experiments **28**

13. VT-ECD Experiments 29

VT-ECD Experiments for poly-(*P*)-**1** 29

VT-ECD Experiments for poly-(*M*)-**1** 32

VT-ECD Experiments for poly-(*P*)-**2** 33

14. Atomic Force Microscopy (AFM) Measurements for poly-(*P*)-1 37

15. Theoretical Calculations 38

16. ECD and UV-vis studies of poly-(*P*)-1 in presence of different anions 42

17. NMR Anion Titration Experiments 44

NMR studies of the mono-(*P*)-**1** in the presence of different anions 45

NMR studies of the poly-(*P*)-**1** in the presence of different anions 47

18. References 48

1. Materials and Methods

All chemicals were purchased as reagent grade and used without further purification. Reactions were conducted in dry solvents under Argon unless otherwise stated.

NMR experiments have been recorded in a Bruker spectromer (^{1}H frequency 300 mHz).

^{1}H NMR STD and NOESY experiments have been measured at 278 K in a Bruker NEO-750 spectrometer (^{1}H frequency 750 mHz).

ECD measurements were done in a Jasco-720 and UV spectra were registered in a Jasco V-750 with a 1 mm quartz cuvette. VT-ECD were measured in a Jasco-1100. The amount of polymer used is indicated in the corresponding section.

ATR/FT-IR spectra were recorded in a Perkin Elmer FT-IR ATR Spectrum Two.

Optical rotation was measured in a Jasco-P2000.

Raman spectra were carried out in a Reinshaw confocal Raman spectrometer (Invia Reflex model) equipped with two lasers (diode laser 785 nm and Ar laser 514 nm).

Chiral HPLC experiments were carried out in a Waters System equipped with a Phenomenex Lux 5 μm i-Amilose-1 column. The amount of monomer used was 0.5 $mg\cdot mL^{-1}$ and the mixture hexane:isopropanol (9:1) was used as eluent (flow rate: 0.5 $mL\cdot min^{-1}$).

GPC studies were carried out in a Waters Alliance 2695 HPLC with a UV-2489 detector (Waters) mns equipped with Phenomenex GPC columns (103 Å, 104 Å and 105 Å). The amount of polymer used was 0.5 mg mL^{-1}. THF was used as eluent (flow rate: 1 $mL\cdot min^{-1}$) and as inner standard, polystyrene narrow standards (PSS) were used.

DSC traces were obtained in a DSC Q200 Tzero Technology (TA Instruments, New Castle, UK), equipped with a refrigerated cooling system RCS90 (TA Instruments, New Castle, UK), using a Tzero low-mass aluminum pan.

TGA traces were obtained in a TGA Q5000 (TA Instruments, New Castle, UK) using a platinium pan.

AFM measurements were performed in a Multimode V Scanning Probe Microscope (Veeco Instruments) in air at rt, with standard silicon cantilevers and supersharp cantilevers in tapping mode using 12 μm and 1 μm scanners. Nanoscope processing software and WSxM 4.0 Beta 1.0 [4] (Nanotec Electrónica, S.L.) was used for image analysis. All measurements were performed at CACTI (University of Vigo, Spain).

PyMOL was used as a molecular visualization system.

Gaussian-16 (G16RevC.01) was used to compute the different molecules.

2. Synthesis of Monomers

Di-tert-butyl-2-methyl-9-(4-((trimethylsilyl)ethynyl)phenyl)nona-5,6-dien-3,8-diyn-2-ol (S1)

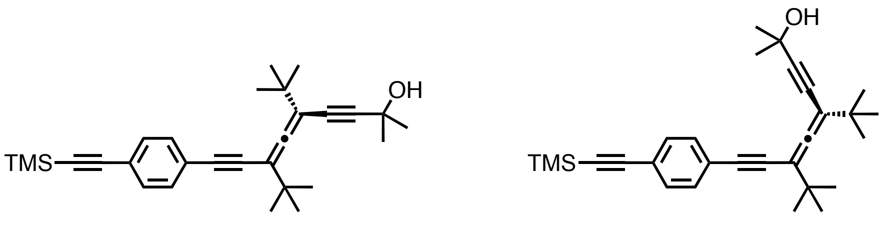

(*P*)-**S1** (*M*)-**S1**

Compounds (*P*)-**S1** and (*M*)-**S1** were prepared according to the procedure described in the reference S1.

Molecular Formula: $C_{29}H_{38}OSi$. **MW:** 430.71 **g/mol.**

^{1}H-NMR (300 MHz, $CDCl_3$, 298K) *δ:* 7.38 (m, 4H), 1.56 (s, 6H), 1.19 (s, 9H), 1.15 (s, 9H), 0.25 (s, 8H).

^{13}C-NMR (75 MHz, $CDCl_3$, 298K) *δ:* 211.7, 132.1, 131.5, 124.1, 122.9, 105.1, 103.6, 103.2, 97.7, 96.4, 92.5, 85.7, 76.1, 66.1, 36.0, 35.9, 31.9, 0.3.

HRMS (ESI$^+$): *m/z* calcd. for $C_{29}H_{38}OSi$ 430.2692; found 431.2758 $[M+H]^+$.

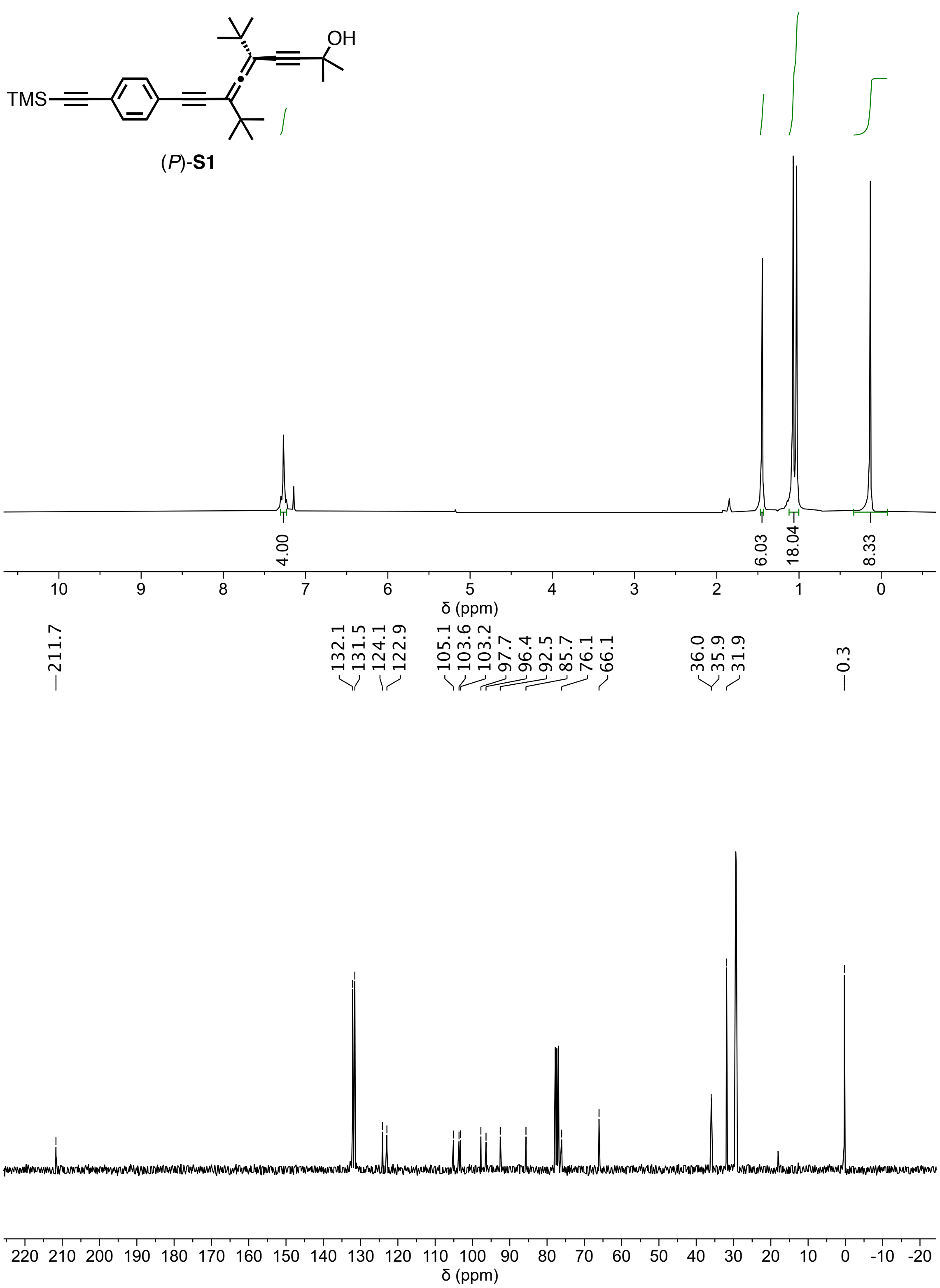

Figure S1. ^{1}H-NMR and ^{13}C-NMR of (*P*)-**S1** in $CDCl_3$.

*(P)-N-(5,7-di-tert-butyl-2-methyl-9-(4-((trimethylsilyl)ethynyl)phenyl)nona-5,6-dien-3,8-diyn-2-yl)-4-methylbenzenesulfonamide (**S2**)*

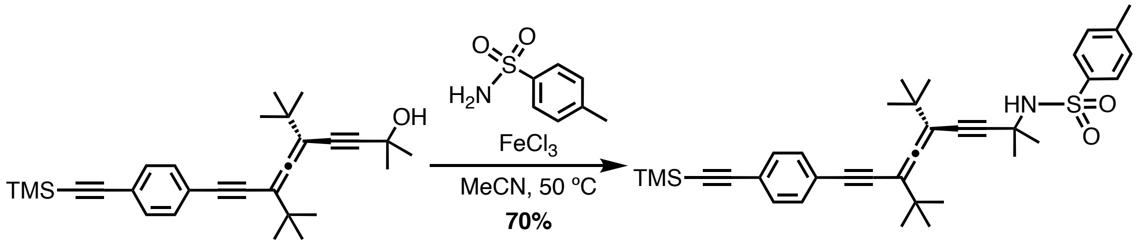

(*P*)-**S1** (*P*)-**S2**

Propargylic alcohol (*P*)-**S1** (100 mol%, 0.313 mmol, 135 mg), *p*-toluenesulfonamide (300 mol%, 0.940 mmol, 161 mg), 650 μL CH_3CN and $FeCl_3$ (5 mol%, 0.016 mmol, 3mg) were successively added to a 5-mL flask, and the reaction mixture was stirred at 50°C overnight. Upon completion, the solvent was concentrated under reduced pressure, and then the residue was purified by silica gel column chromatography (Hexane:EtOAc (8.5:1.5)) to afford (*P*)-**S2** as a yellow oil (128 mg, 70%).

Molecular Formula: $C_{36}H_{45}NO_2SSi$. **MW:** 583.906 **g/mol.**

^{1}H-NMR (300 MHz, CD_3CN, 298K) *δ:* 7.73(d, 2H), 7.45 – 7.38 (m, 4H), 7.32 (d, 2H), 5.88 (s, 1H), 2.31 (s, 3H), 1.52 (s, 3H), 1.49 (s, 3H), 1.16 (s, 9H), 1.01 (s, 9H), 0.22 (s, 9H).

^{13}C-NMR (75 MHz, CD_3CN, 298K) *δ:* 212.1, 144.00 140.8, 132.8, 132.4, 130.4, 128.0, 124.5, 123.8, 105.3, 104.3, 97.3, 96.7, 93.1, 86.1, 77.0, 51.4, 36.4, 36.3, 31.8, 31.5, 29.3, 29.1, 21.6, -0.1.

HRMS (ESI$^+$): *m/z* calcd. for $C_{36}H_{45}NO_2SSi$ 583.2940; found 584.3013 $[M+H]^+$.

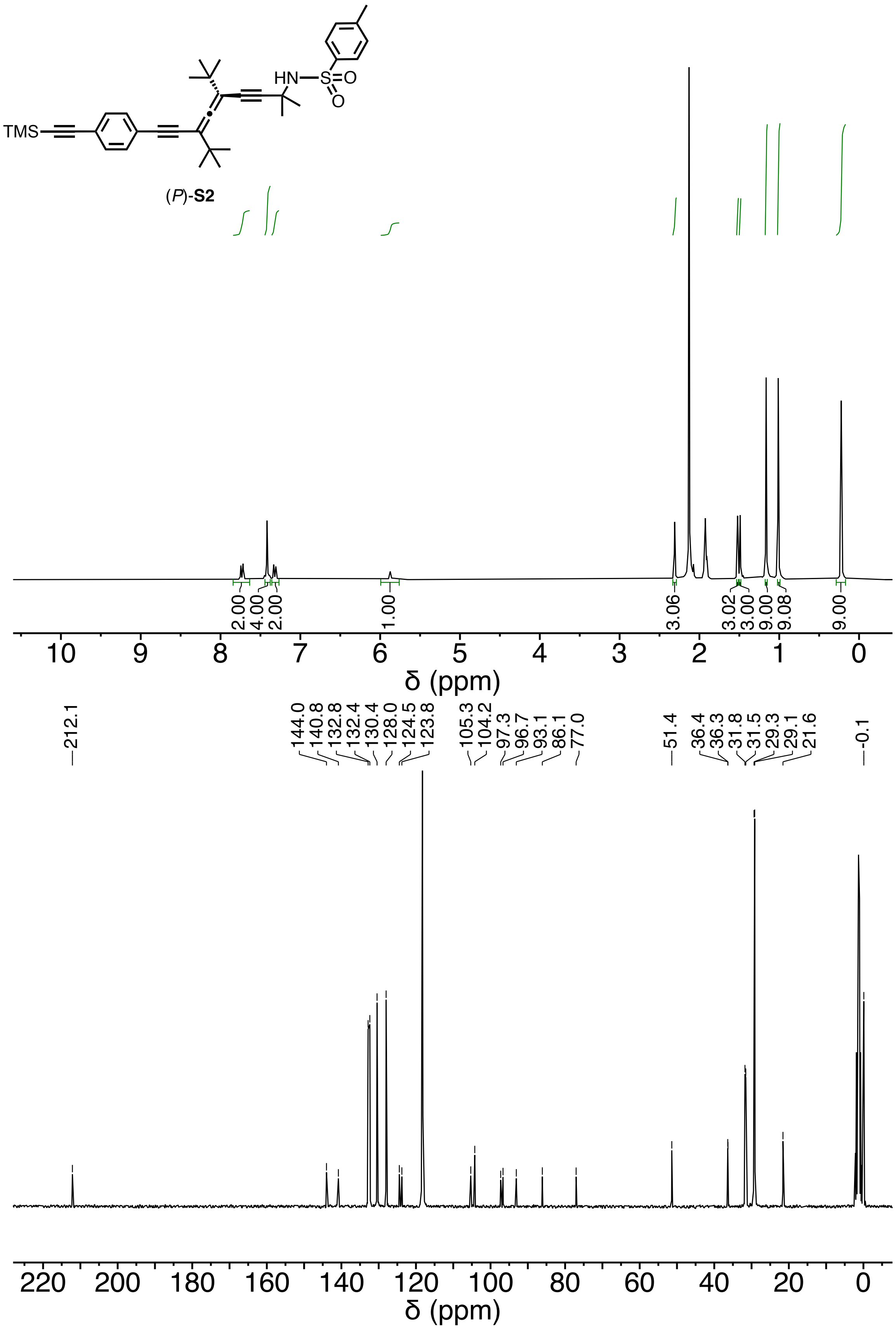

Figure S2. ^{1}H-NMR and ^{13}C-NMR of (*P*)-**S2** in CD_3CN.

*(P)-Di-tert-butyl-9-(4-ethynylphenyl)-2-methylnona-5,6-dien-3,8-diyn-2-ol (mono-(P)-**1**)*

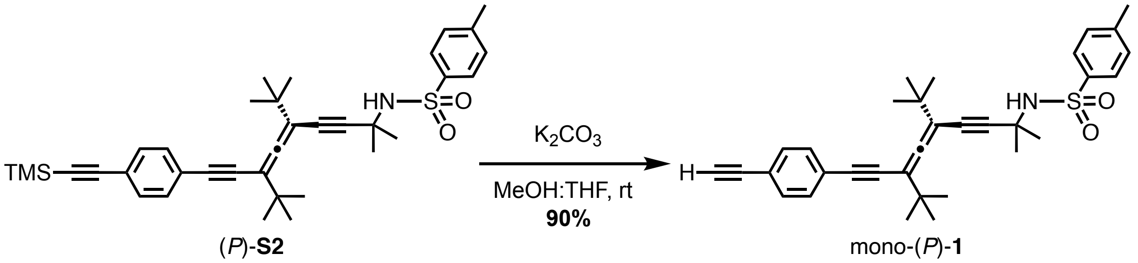

To a solution of (*P*)-**S2** (100 mol%, 0.197 mmol, 115 mg) in a mixture of THF:MeOH (1:1, 4 mL), K_2CO_3 (300 mol%, 0.591 mmol, 82 mg) was added at rt. After 30 min, the mixture was quenched with distilled water (20 mL) and then the aqueous phase was extracted with DCM (3 x 10 mL). The combined organic phases were dried over anhydrous Na_2SO_4. Evaporation *in vacuo* and purification by flash chromatography on silica gel (hexane: EtAcO (8.5:1.5) afforded mono-(*P*)-**1** (91 mg, 90%) as a yellow oil.

For mono-(*P*)-**1**, $[\alpha]_D^{20}$= +302 (*c* = 10 mg·mL^{-1}, $CHCl_3$)

Molecular Formula: $C_{33}H_{37}NO_2S$. **MW:** 511.72 **g/mol.**

^{1}H-NMR (300 MHz, CD_3CN) *δ* 7.74 (d, 2H), 7.49 – 7.43 (m, 4H), 7.33 (d, 2H), 5.86 (s, 1H), 3.50 (s, 1H), 2.31 (s, 3H), 1.52 (s, 3H), 1.49 (s, 3H), 1.17 (s, 9H), 1.03 (s, 9H).

^{13}C-NMR (75 MHz, CD_3CN) *δ* 212.2, 144.1, 140.9, 133.2, 132.4, 130.5, 124.8, 123.0, 118.3, 104.30, 104.3, 96.7, 93.1, 86.1, 83.8, 80.9, 77.1, 51.5, 36.4, 36.4, 31.8, 31.6, 29.4, 29.2, 21.6.

HRMS (ESI$^+$): *m/z* calcd. for $C_{33}H_{37}NO_2S$ 511.2545; found 512.2623 $[M+H]^+$.

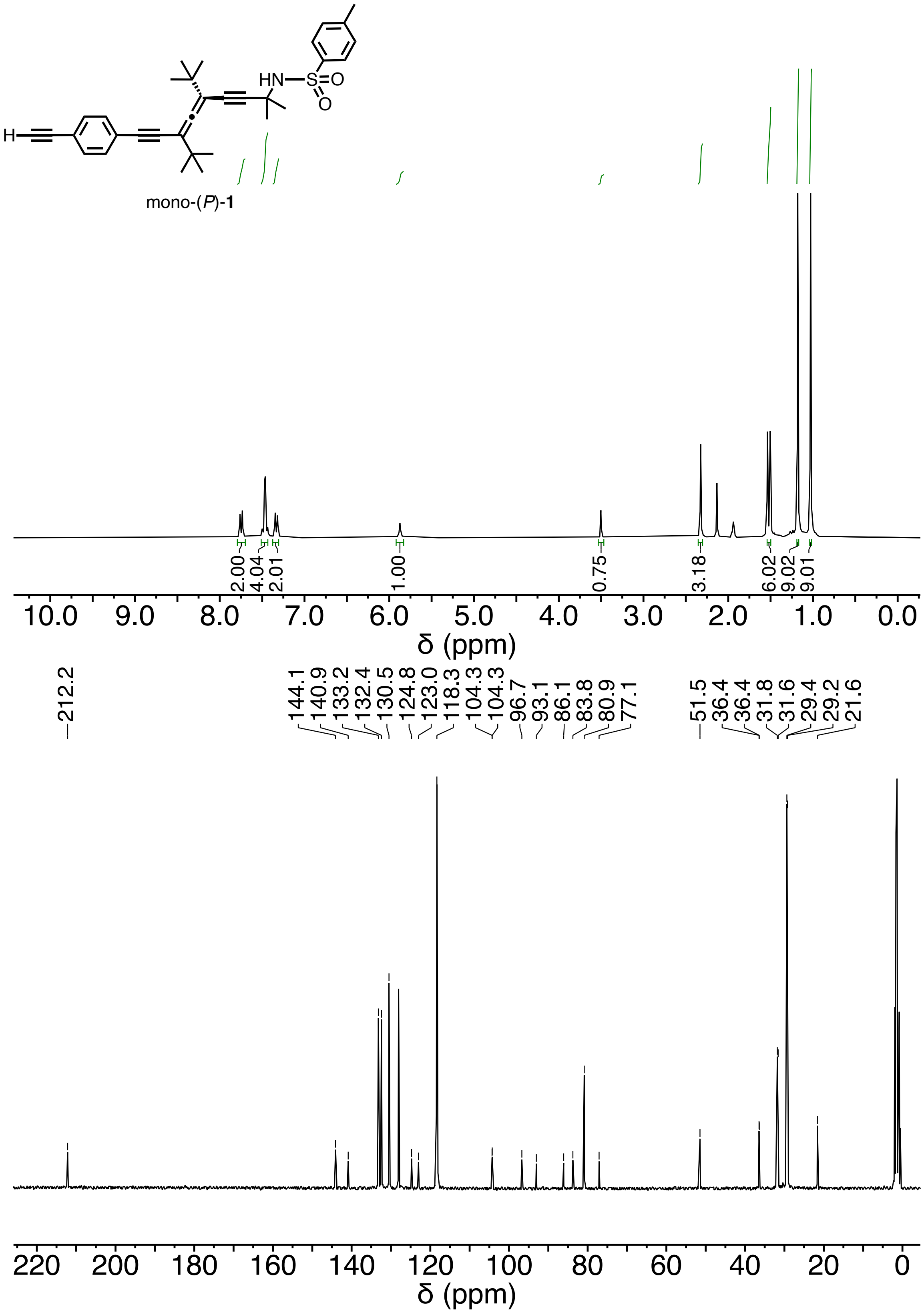

Figure S3. ^{1}H-NMR and ^{13}C-NMR of mono-(*P*)-**1** in CD_3CN.

(M)-Di-tert-butyl-9-(4-ethynylphenyl)-2-methylnona-5,6-dien-3,8-diyn-2-ol (mono-*(M)*-**1***)*

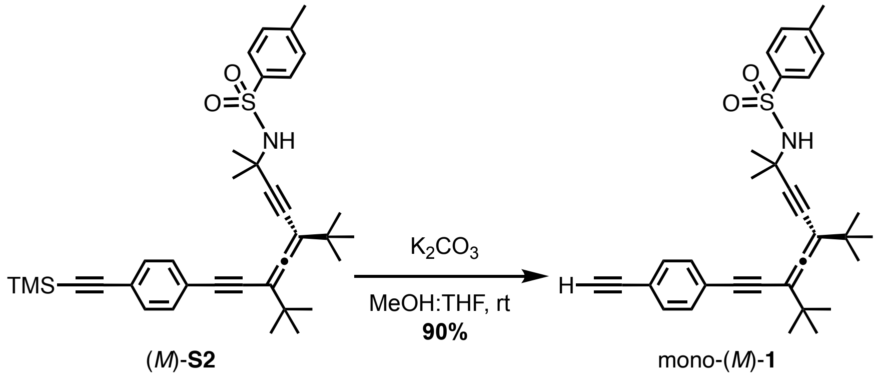

Enantiomer mono-(*M*)-**1** was obtained from (*M*)-**S2** by applying the same protocol as for mono-(*P*)-**1**. For mono-(*M*)-**1** $[\alpha]_D^{20}$= -316 (*c* = 10 mg·mL^{-1}, $CHCl_3$)

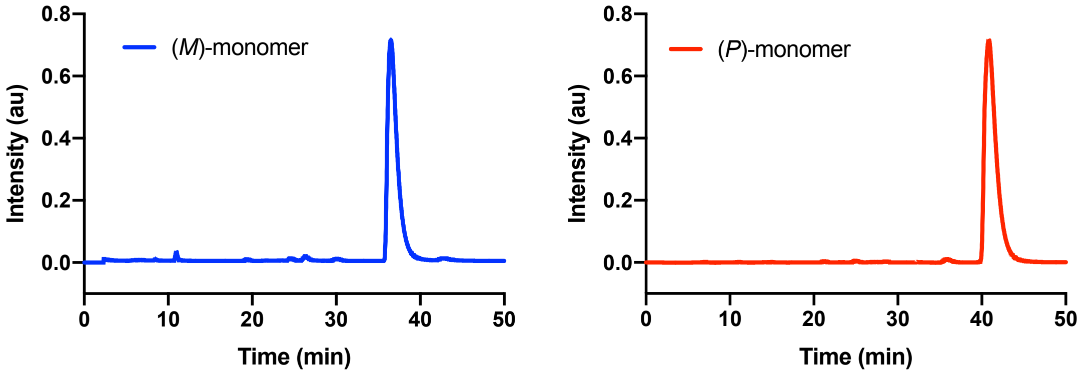

Figure S4. Chiral HPLC traces for mono-(*M*)-**1** and mono-(*P*)-**1** respectively (ee> 98%).

*(M)-N-(5,7-di-tert-butyl-9-(4-ethynylphenyl)-2-methylnona-5,6-dien-3,8-diyn-2-yl)-N,4-dimethylbenzenesulfonamide (*mono-*(P)*-**2***)*

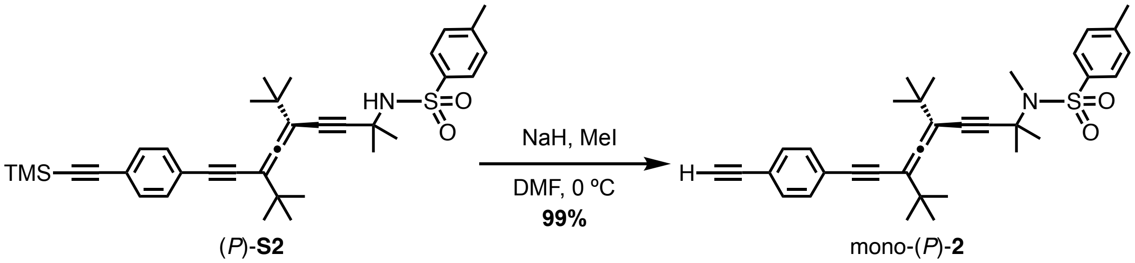

A pre-cooled solution (0 °C) of (*P*)-**S2** (100 mol%, 0.103 mmol, 60 mg) in dry DMF (1 mL) was treated with NaH (powder moistened with oil, 55–65%) (220 mol%, 0.226 mmol, 9 mg). After 45 min, MeI (2000 mol%, 2.060 mmol, 0.1 mL) was added. The resulting mixture was stirred for 10 min at 0 °C. The reaction mixture was quenched with sat. aq. NH_4Cl (10 mL) and then the aqueous phase was extracted with DCM (3 x 10 mL). The combined organic phases were washed with 5% aq. LiCl, dried over anhydrous Na_2SO_4. Evaporation *in vacuo* and purification by flash chromatography on silica gel (hexane: EtAcO (9:1) afforded mono-(*P*)-**2** (54 mg, 99%) as a yellow oil.

For mono-(*P*)-**2**, $[\alpha]_D^{20}$= +322 (*c* = 10 mg·mL^{-1}, $CHCl_3$)

Molecular Formula: $C_{34}H_{39}NO_2S$. **MW:** 525.75 **g/mol.**

^{1}H-NMR (300 MHz, CD_3CN) δ 7.70 (d, 2H), 7.49 – 7.40 (m, 4H), 7.33 (d, 2H), 3.49 (s, 1H), 3.01 (s, 3H), 2.33 (s, 3H), 1.64 (s, 3H), 1.62 (s, 3H), 1.16 (s, 9H), 1.03 (s, 9H).

^{13}C-NMR (75 MHz, CD_3CN) δ 212.2, 144.4, 139.5, 133.1, 132.3, 130.6, 128.2, 124.7, 122.9, 104.3, 104.2, 96.2, 93.0, 85.9, 83.7, 80.8, 77.9, 56.9, 36.4, 36.2, 34.5, 30.6, 30.6, 29.3, 29.1, 21.5.

HRMS (ESI$^+$): *m/z* calcd. for $C_{34}H_{39}NO_2S$ 525.2702; found 548.2595 $[M+Na]^+$.

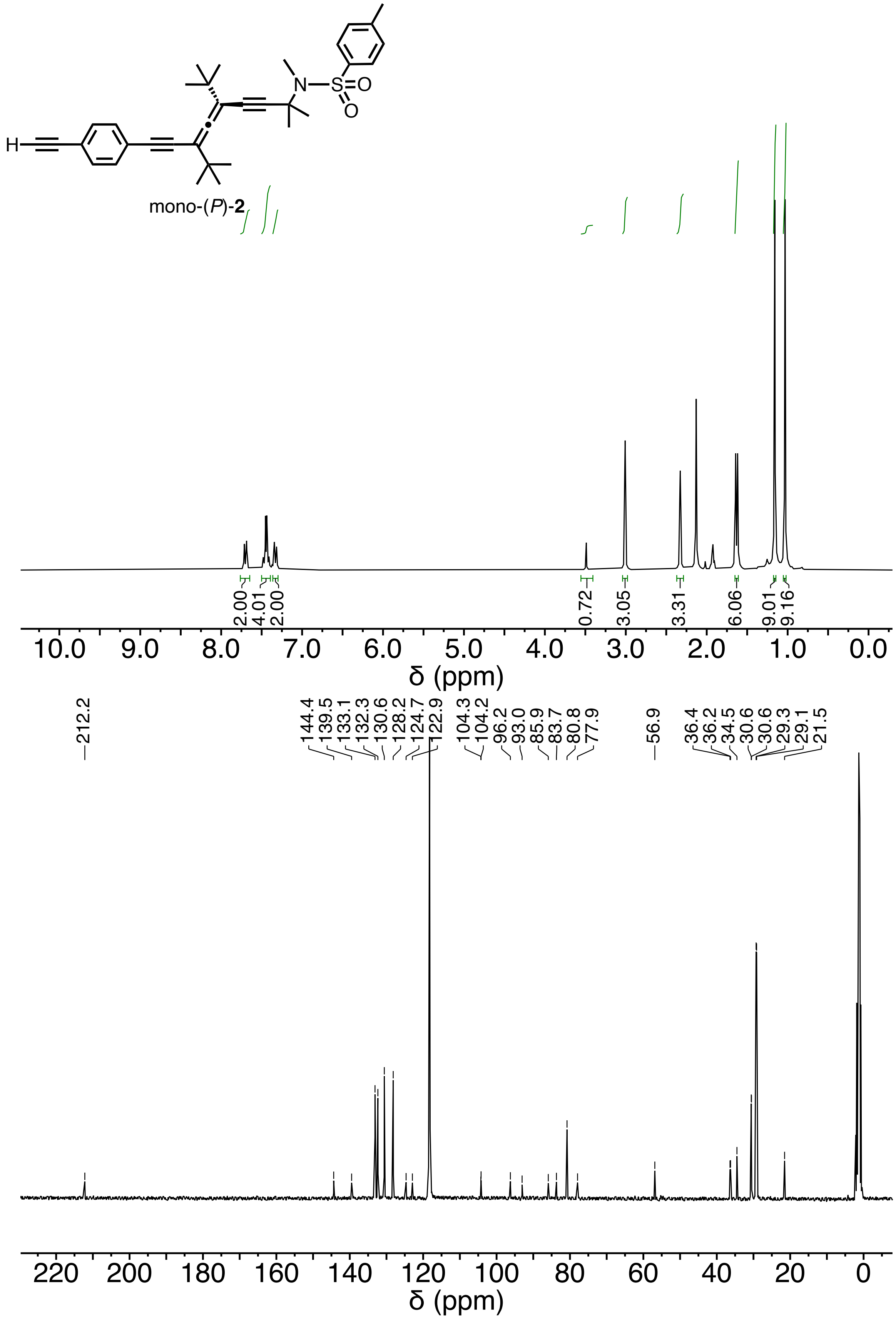

Figure S5. ^{1}H-NMR and ^{13}C-NMR of mono-(*P*)-**2** in CD_3CN.

3. Synthesis of polymers

General procedure for polymerization

The reaction flask (sealed ampoule) was dried under vacuum and argon flushed for three times before a solution of monomer in THF was added *via* cannula. Then, triethylamine was added dropwise *via* syringe. A solution of rhodium norbornadiene chloride dimer, $[Rh(nbd)Cl]_2$, in THF was added under stirring at rt. The reaction mixture was stirred at rt overnight. Then, the resulting polymer was diluted in DCM and precipitated in a large amount of MeOH, centrifuged (2 times), reprecipitated in hexane and centrifuged again (Table S1).

Table S1. Calculated amounts for the synthesis of the polymers poly-(*P*)-**1** and poly-(*M*)-**1**.

Monomer	Mass (mg)	THF (μL)	Et_3N (μL)	Catalyst (mg)	Yield (%)
mono-(*P*)-**1**	67	300	4	1	85
mono-(*M*)-**1**	50	200	3	1	83
mono-(*P*)-**2**	50	200	3	1	88

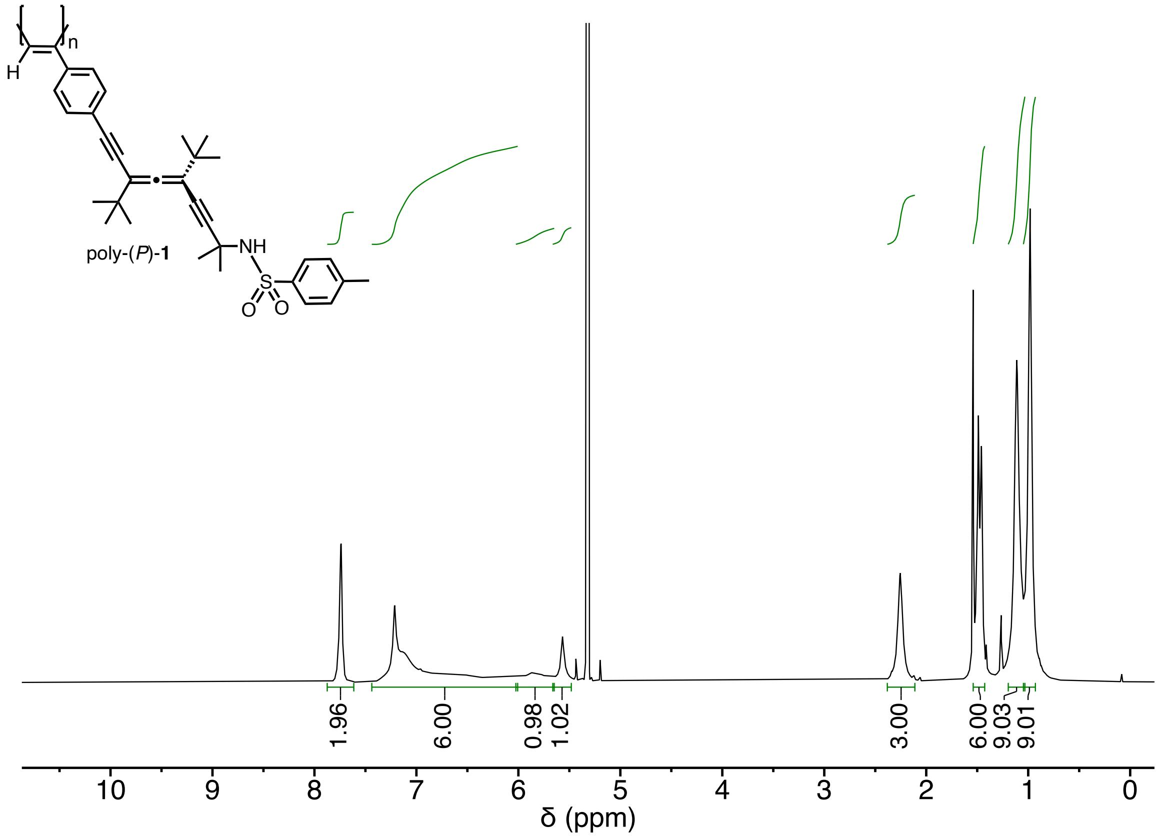

Figure S6. ^{1}H-NMR of poly-(*P*)-**1** in CD_2Cl_2.

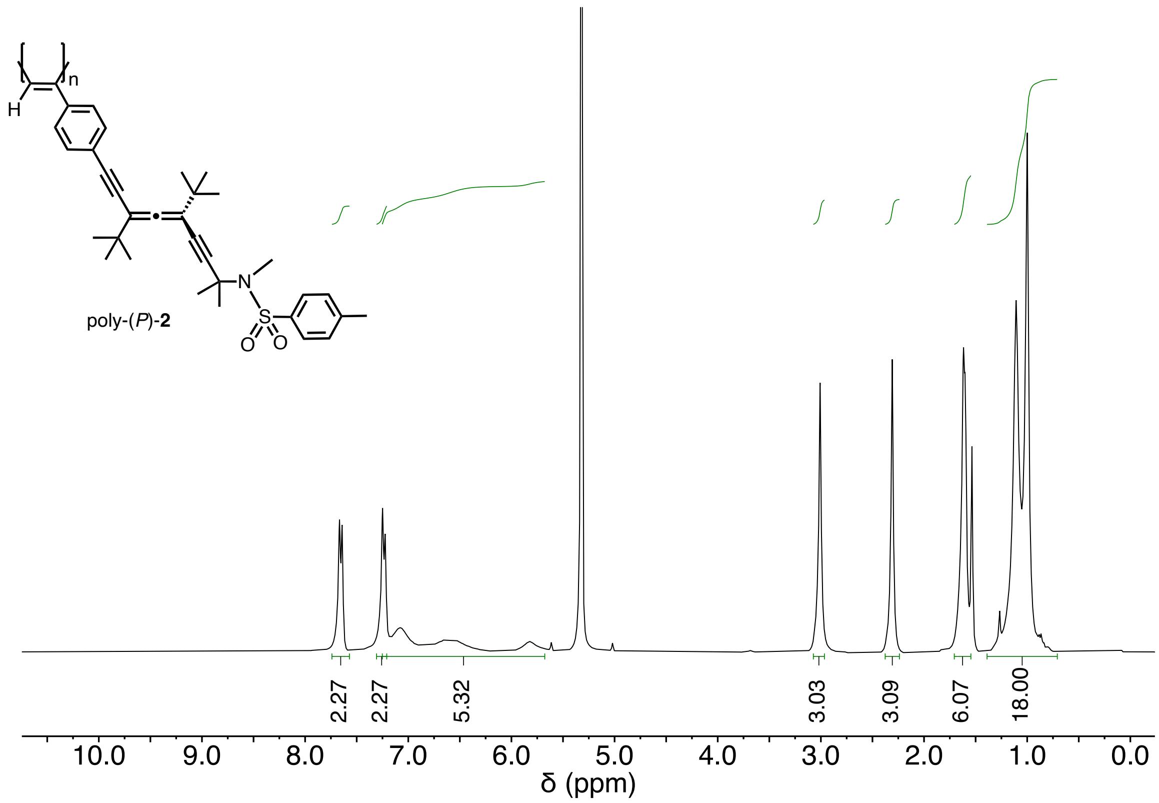

Figure S7. ^{1}H-NMR of poly-(*P*)-**2** in CD_2Cl_2.

4. GPC Studies

The molecular weight was estimated by GPC using THF (flow rate: 1.0 mL·min^{-1}) as eluent and polystyrene narrow standards (PSS) as calibrants.

Table S2. GPC data for the synthesized polymers.

Polymer	Mn (Daltons)	Mw (Daltons)	Mz (Daltons)	Đ (Daltons)
poly-(*P*)-**1**	121887	182669	2303938	1.37
poly-(*M*)-**1**	97854	121555	160857	1.24
poly-(*P*)-**2**	166513	204953	240085	1.23

5. Raman Experiments

The bands observed by Raman resonance confirmed the former configuration. The peak at highest wavelength corresponds to the C=C bond stretching and overlaps with that of the phenyl ring. The band at 1334 cm^{-1} arises from the *cis* C-C bond coupled with the single bond connecting the main chain and the phenyl ring. The peak at lowest wavelength (915 cm^{-1}) corresponds to the C-H bond of the *cis* form. The disappearance of the alkyne peak (ca. 2106 cm^{-1}) also confirms the formation of the polymer.

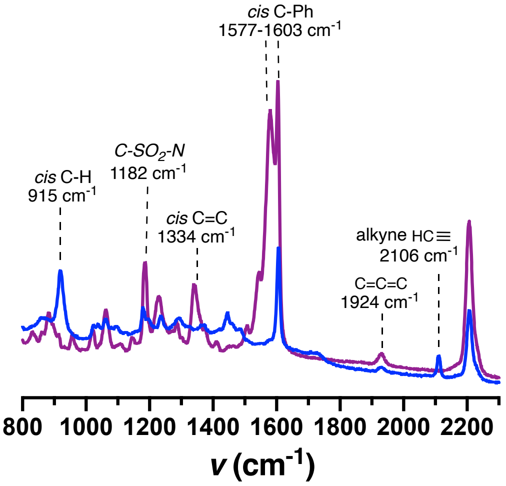

Figure S8. Raman spectra of mono-(*P*)-**1** and poly-(*P*)-**1**.

6. Thermal Studies

DSC Studies

DSC experiments have been used to determine the configuration of the polymer skeleton in poly(phenylacetylene)s due to the different thermogram pattern for a *cis*-cisoidal (*c-c*) or a *cis*-transoidal (*c-t*) polyene backbone.[S2,S3] Hence, the geometry of the polymer was determined by DSC. According to a general protocol, the polymer was placed in an aluminum pan up to 400 °C (heating rate: 10 °C.min^{-1}). The thermogram for poly-(*P*)-**1** shows a typical *c-t* trace with an exothermal peak, at 102 °C and 185 °C respectively, correspondent to a *c-c* transition and a final isomerization to *t-t* (Figure S9).

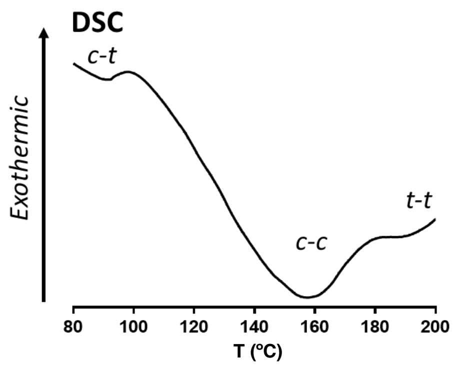

Figure S9. DSC study for poly-(*P*)-**1**.

TGA studies

The thermal stability was evaluated by TGA. As a general protocol, the polymer sample was placed in a platinum pan and heated from 40°C to 800°C (heating rate: 10 °C·min^{-1}). Poly-(*P*)-**1** starts to degrade at 280 °C (Figure S10).

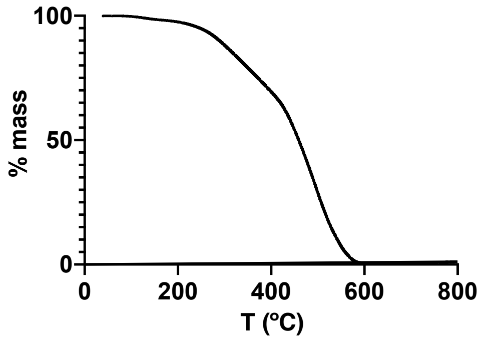

Figure S10. TGA thermogram for poly-(*P*)-**1**.

7. ECD Studies of mono-(*M*)-1

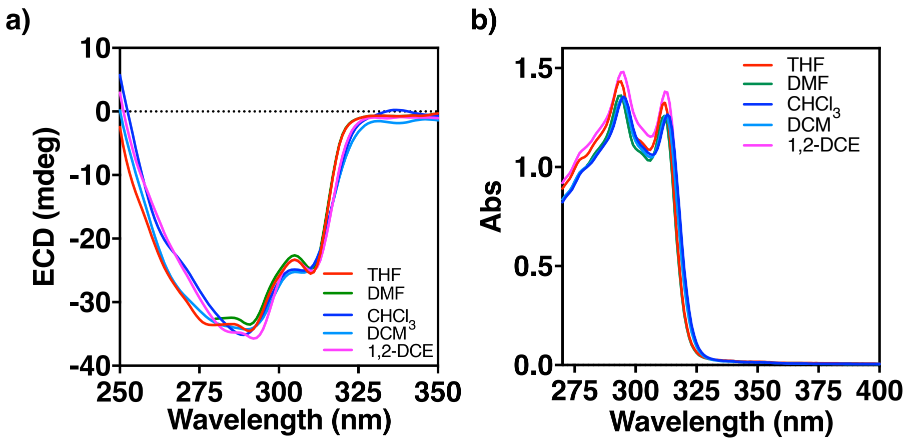

Figure S11. a) ECD spectra and UV-vis (b) of mono-(*M*)-**1** in different solvents (0.8 mM).

8. Studies of the effect of Lewis base and non-Lewis base solvents on the conformation of mono-(*P*)-1 by NMR

*^{1}H-NMR studies of mono-(P)-**1** in non-Lewis base and Lewis base solvents*

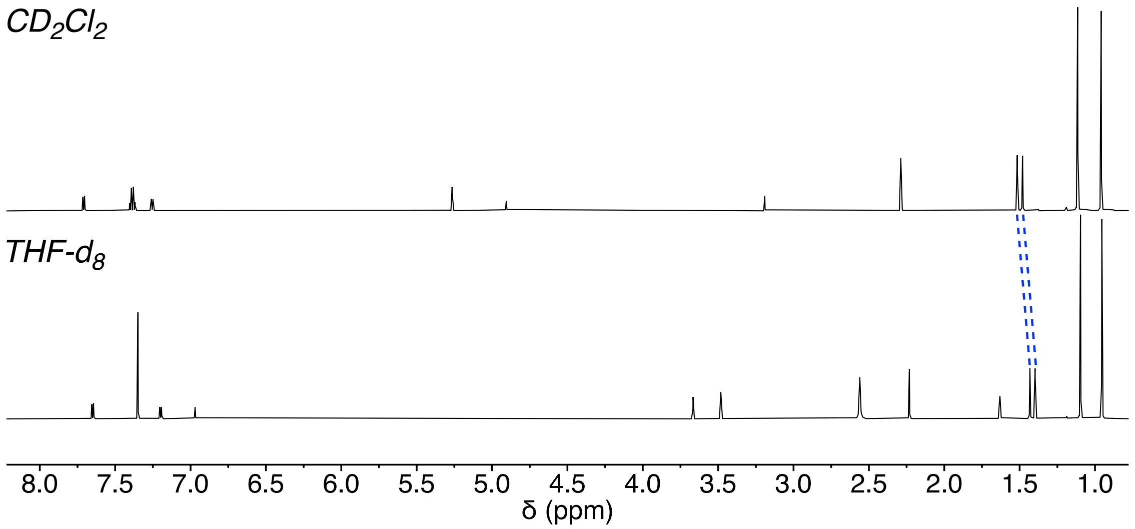

Figure S12. ^{1}H-NMR (750 MHz) of mono-(*P*)-**1** in CD_2Cl_2 (top) and THF-d_8 (below).

*NOESY-NMR studies of mono-(P)-**1** in Lewis base and non-Lewis base solvents*

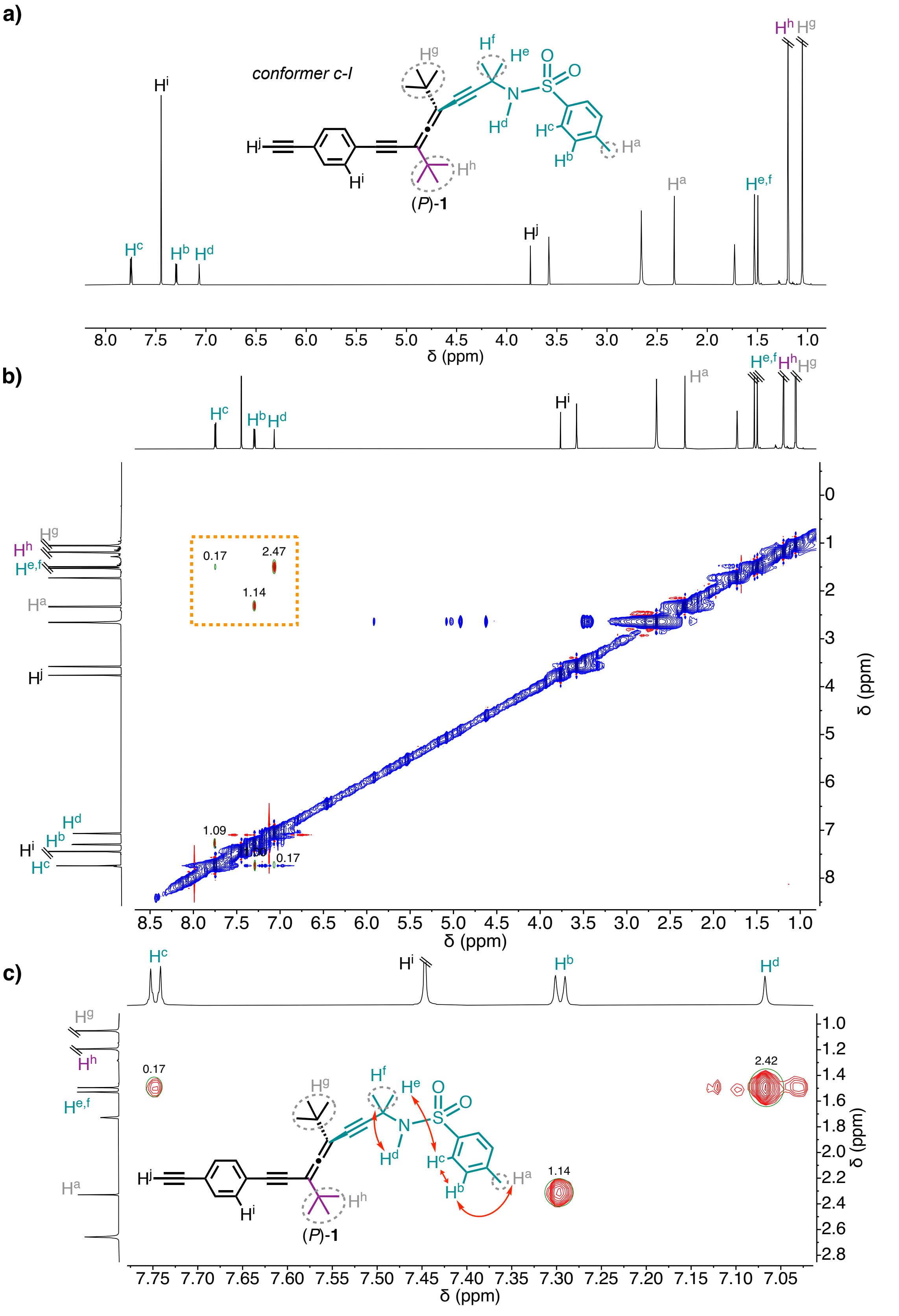

Figure S13. ^{1}H-NMR of mono-(P)-**1** (a) and NOESY (b) and NOESY zoomed area (c) (THF-d$_8$, 278 K, 750 MHz).

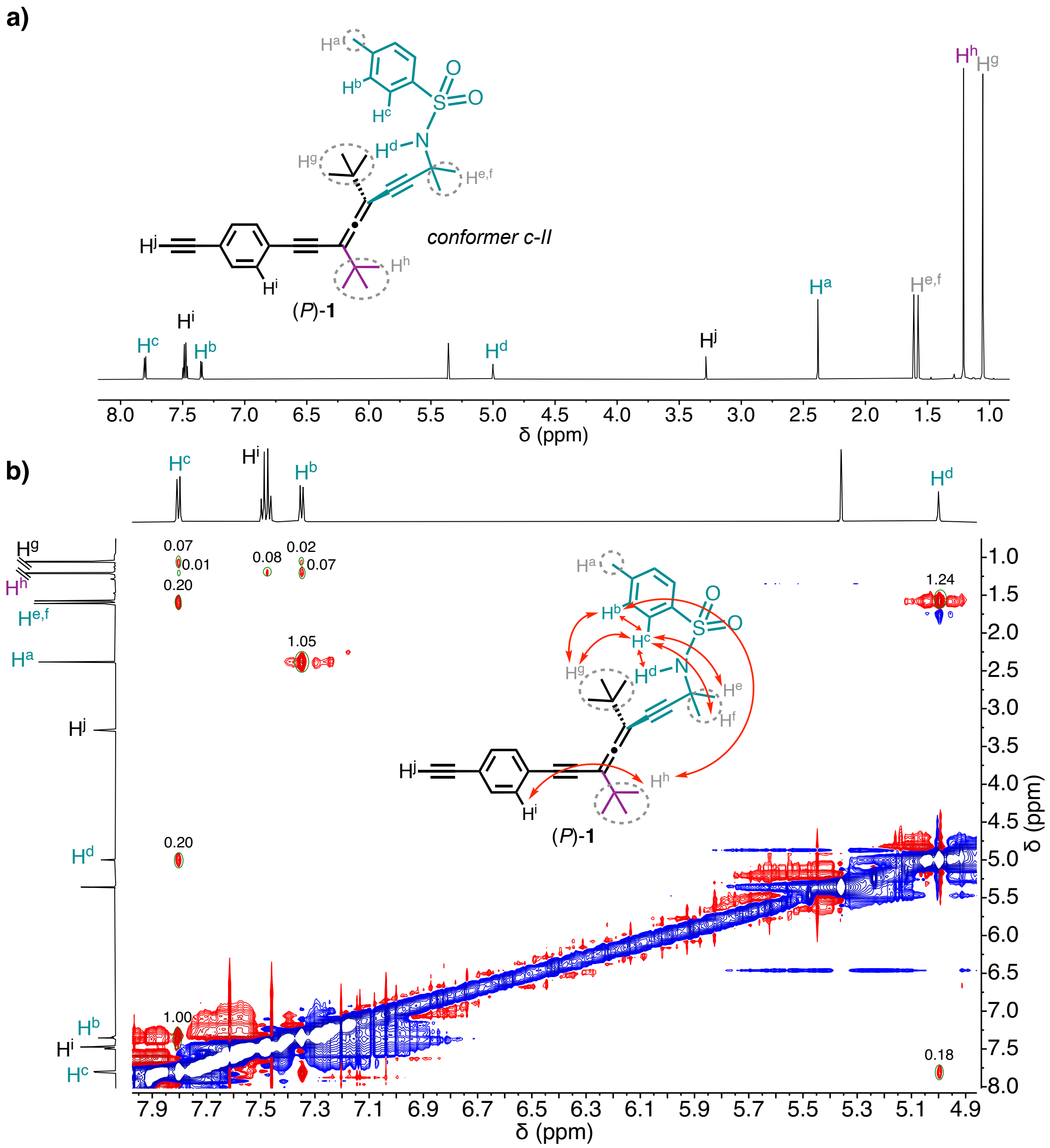

Figure S14. ^{1}H-NMR of mono-(*P*)-**1** (a) and NOESY (b) (CD_2Cl_2, 278 K, 750 MHz).

From these studies it was possible to obtain the distance between the anilide proton with the two methyl groups (Equation 1) of the carbon linked to the sulfonamide group (H^d-$H^{e,f}$), in addition to the distance of these two methyl groups with the *p*-tolyl group ($H^{e,f}$-H^c). Thus, in Lewis base solvent, the *p*-tolyl-sulfonamide group is oriented opposite to the alkyne group (Figure S13, conformer c-I), whereas in non-Lewis base solvents, a bended conformation is generated placing the *p*-tolyl-sulfonamide group closer to the alkyne (Figure S14, conformer c-II).

$$\frac{I_{AB}}{I_{CD}} = \frac{(d_{CD})^6}{(d_{AB})^6}$$

Equation 1. Relationship between interproton distance and NOESY intensities.

Table S3. Interproton distance from NOESY intensities.

Distance (Å)	H^a-H^b	H^c-$H^{e,f}$	H^d-$H^{e,f}$	H^d-H^c	H^b-H^g	H^c-H^g
conformer c-I	2.60	4.08	2.68	3.5	6.41	3.98
conformer c-II	2.60	4.2	2.69	3.4	-	-

9. ECD Studies of polymers

*ECD Studies of poly-(P)-**1***

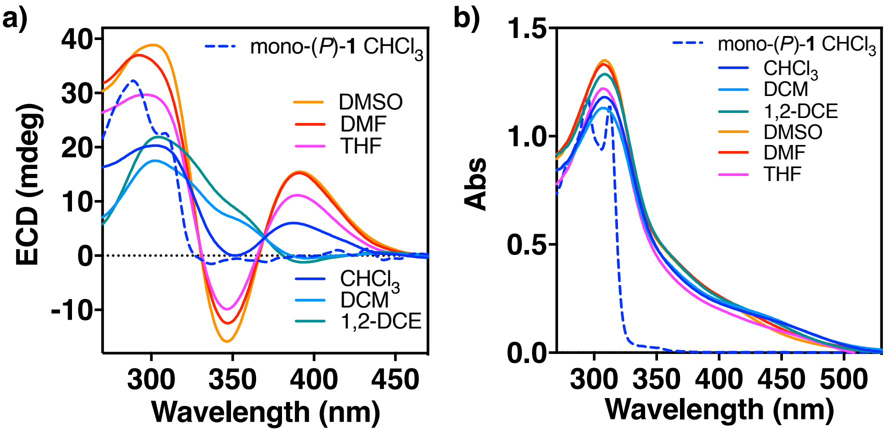

Figure S15. a) ECD spectra and UV-vis (b) of mono-(*P*)-**1** in $CHCl_3$ and poly-(*P*)-**1** in different solvents (0.8 mM).

*ECD Studies of poly-(M)-**1***

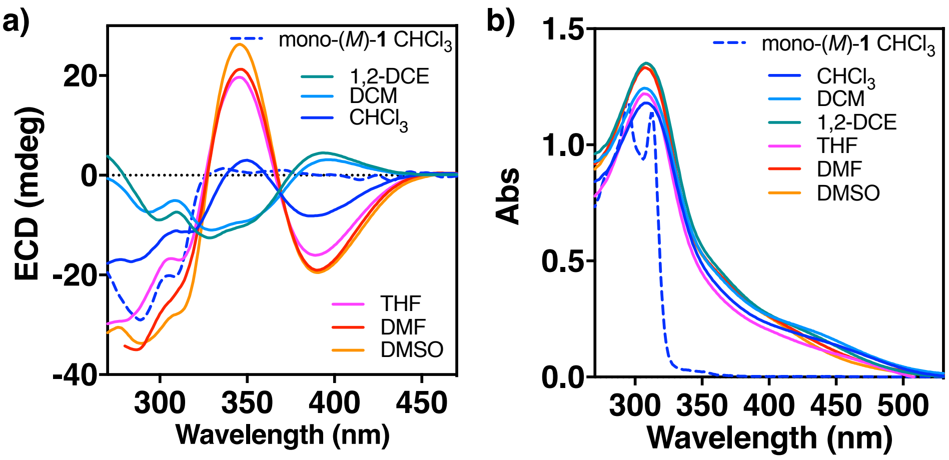

Figure S16. a) ECD spectra and UV-vis (b) of mono-(*M*)-**1** in $CHCl_3$ and poly-(*M*)-**1** in different solvents (0.8 mM).

Comparison of (P)- and (M)-enantiomers

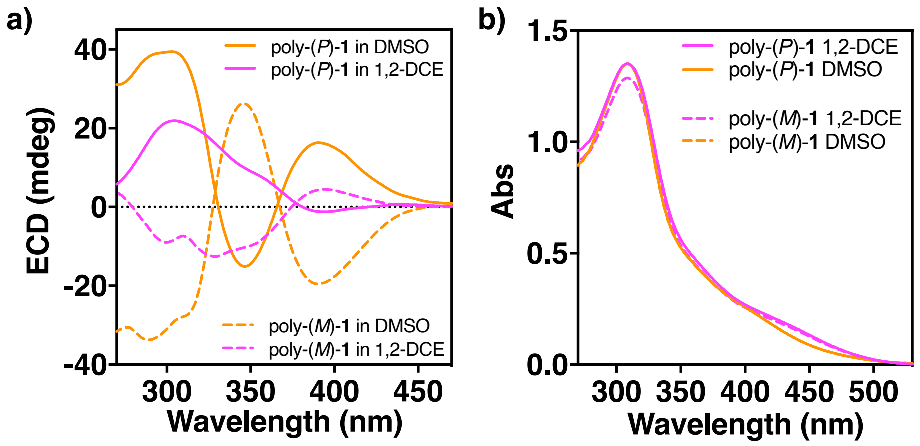

Figure S17. a) ECD spectra comparison of poly-(*P*)-**1** and poly-(*M*)-**1** in different solvents (0.8 mM).

*ECD Studies of poly-(P)-**2***

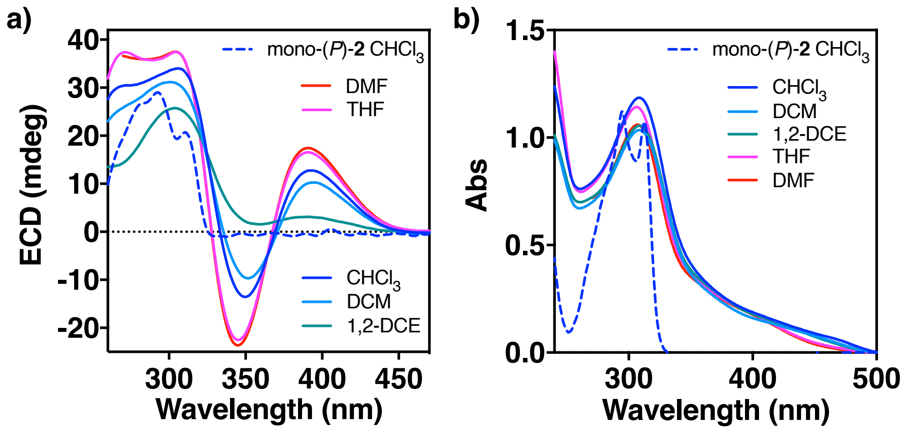

Figure S18. a) ECD spectra and UV-vis (b) of mono-(*P*)-**2** in $CHCl_3$ and poly-(*P*)-**2** in different solvents (0.8 mM).

10. ATR/FT-IR Studies

A solution of poly-(*P*)-**1** (0.8 mM) was prepared in THF and 1,2-DCE, respectively. 20µL of these solutions were placed on a CsI cell and the solvent was removed by evaporation. FT-IR experiments show a shift towards higher frequencies at the characteristic C-SO_2-N stretching vibration in THF (Lewis base solvent) relative to the signal in 1,2-DCE (non-Lewis base solvent)(Figure S19).

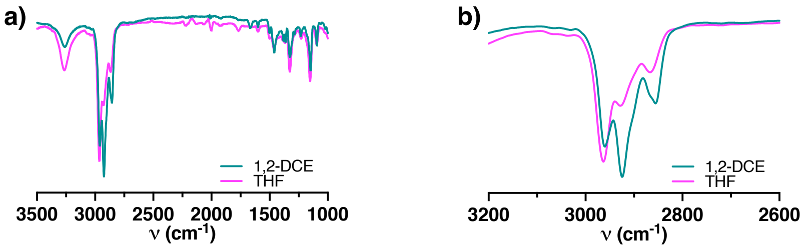

Figure S19. a) IR spectra of a solution of poly-(*P*)-**1** in 1,2-DCE (pink line) and THF (red line) (0.8 mM) and zoomed area relative to the NH of the sulfonamide group (b). FTIR (THF, ν, cm^{-1}): 3276 (NH); 1329,1154 (SO_2). FTIR (1,2-DCE, ν, cm^{-1}): 3245 (NH); 1321, 1146 (SO_2).

Table S4. FT-IR data of poly-**1** in solution relative to the NH of the sulfonamide group.

	ν_{NH} (cm^{-1})	$\Delta\nu_{NH}$ (cm^{-1})
poly-(*P*)-**1** in THF	3276	31
poly-(*P*)-**1** in 1,2-DCE	3245	

11. Studies of the effect of Lewis base and non-Lewis base solvents on the conformation of poly-(*P*)-1 by NMR

*STD-NMR studies of the poly-(P)-**1** in Lewis base solvents and non-Lewis base solvents*

Saturation transfer difference NMR (STD-NMR) experiments were carried out at low temperature (278 K) to study the solvent-polymer interaction to have more precise information about the conformation of the pendants. The results show that, in Lewis base solvent such as THF-d_8, they present STD effects that increase with higher saturation time when the protons of the solvent are saturated. Therefore, Lewis base solvents coordinate with poly-(*P*)-**1** (Figure S20). On the other hand, when the same experiment was carried out using a non-Lewis base solvent such as CD_2Cl_2, no STD effect was observed (Figure S21).

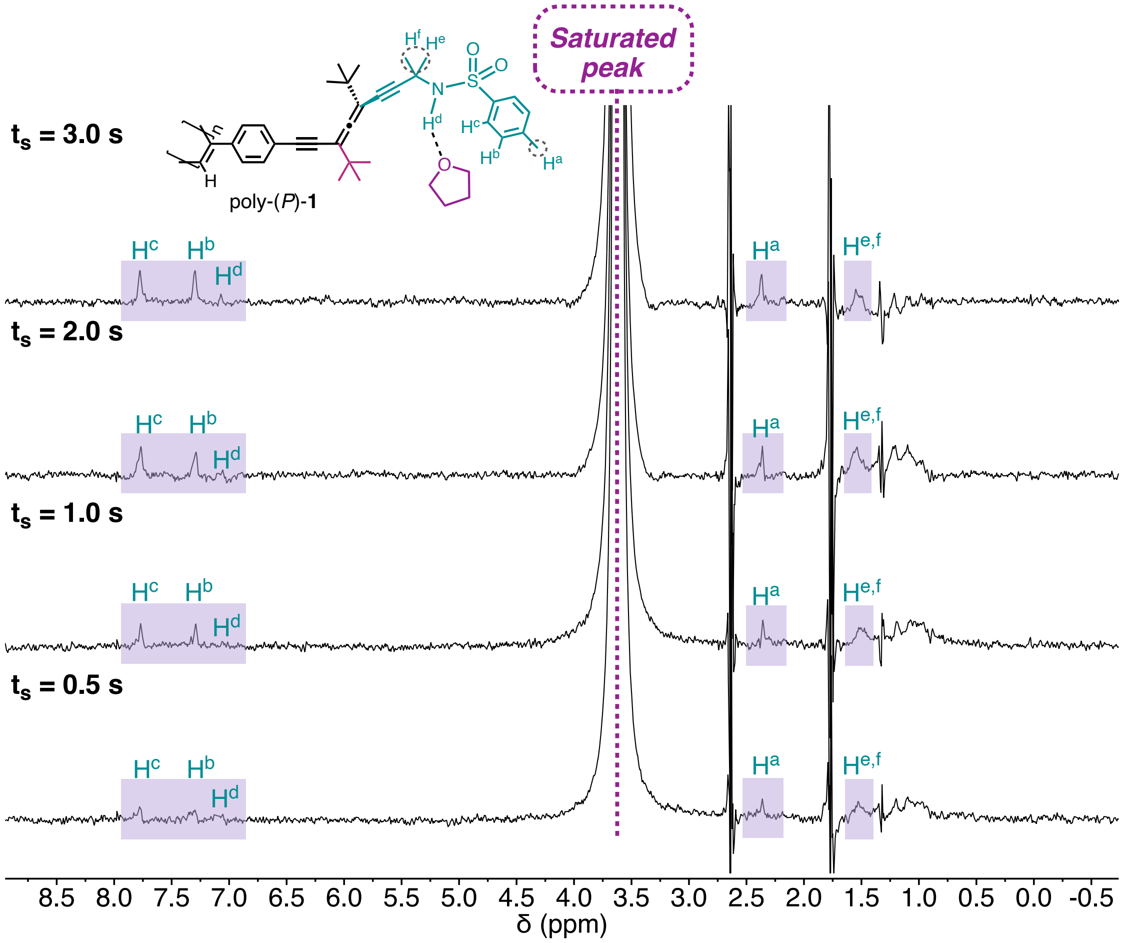

Figure S20. STD-NMR experiment of poly-(*P*)-**1** (THF-d_8, 278 K, 750 MHz). Saturated peak 3.62 ppm.

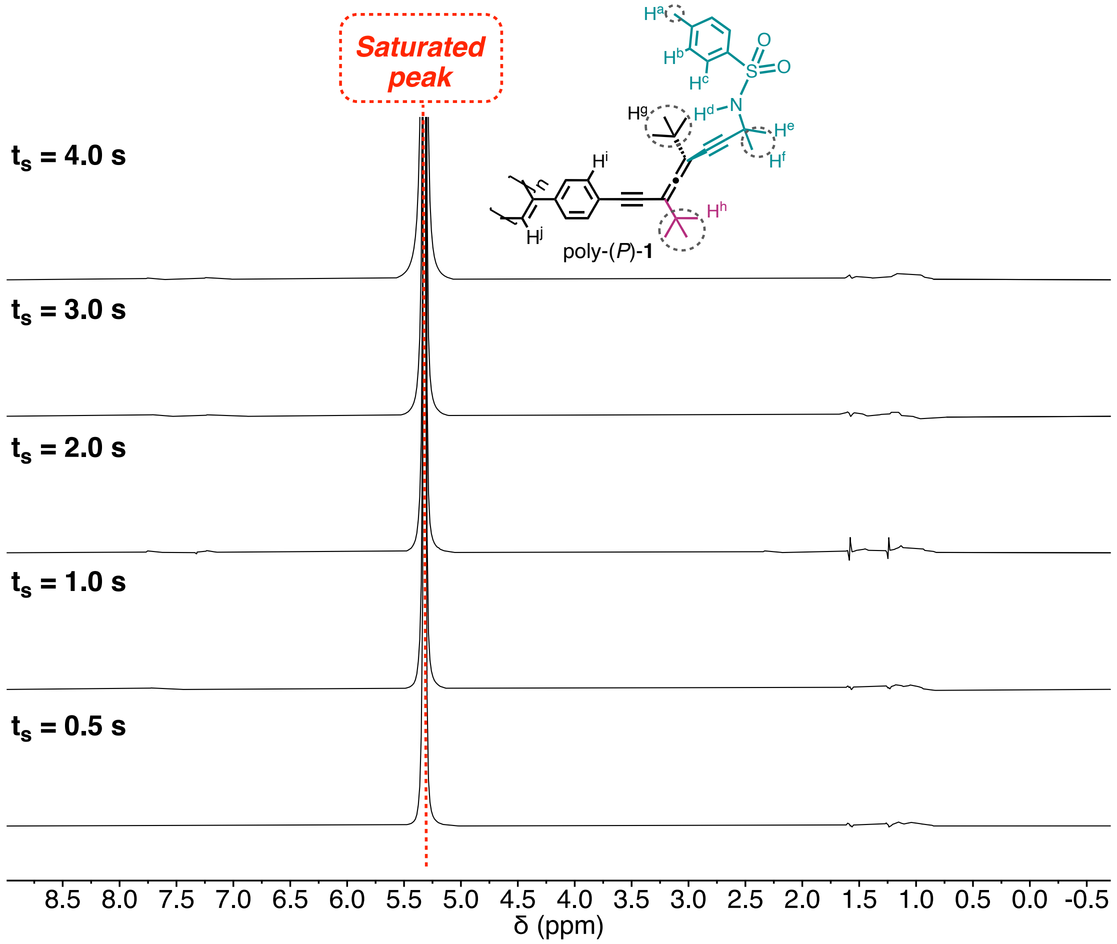

Figure S21. STD-NMR experiment of poly-(*P*)-**1** (CD_2Cl_2, 278 K, 750 MHz). Saturated peak 5.36 ppm.

*NOESY-NMR studies of the poly-(P)-**1** in Lewis base and non-Lewis base solvents*

a)

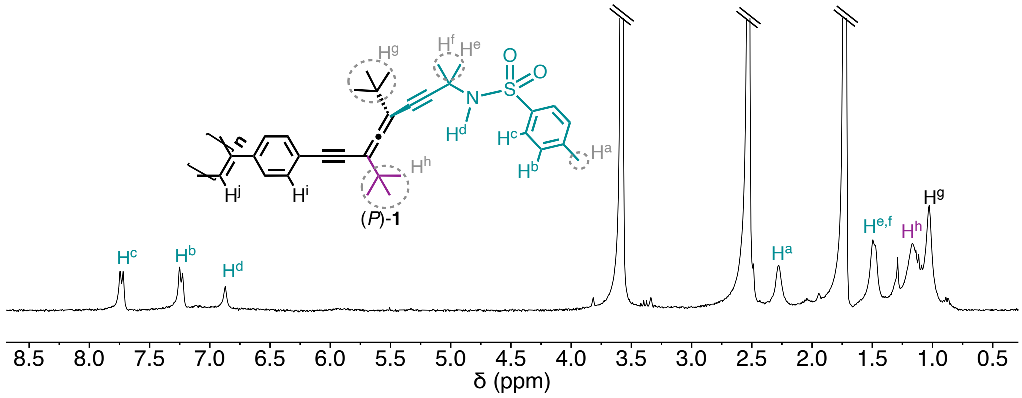

b)

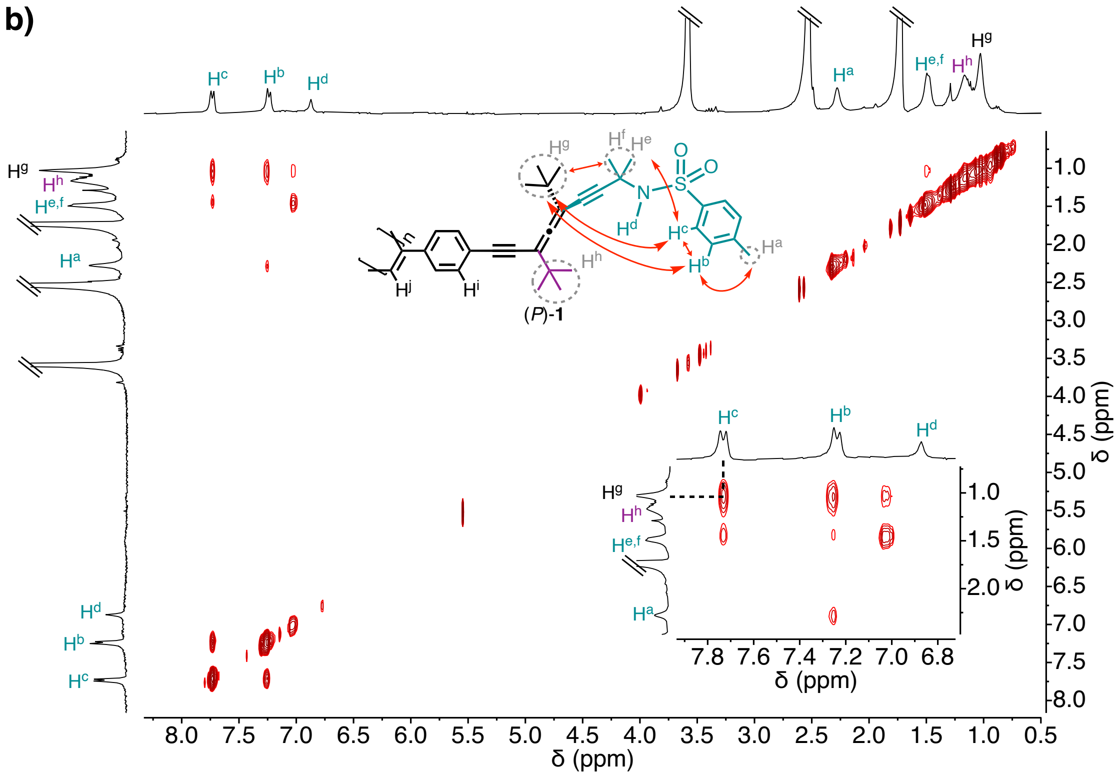

Figure S22. ^{1}H-NMR of poly-(*P*)-**1** (top) and NOESY (below) (THF-d_8, 278 K, 750 MHz).

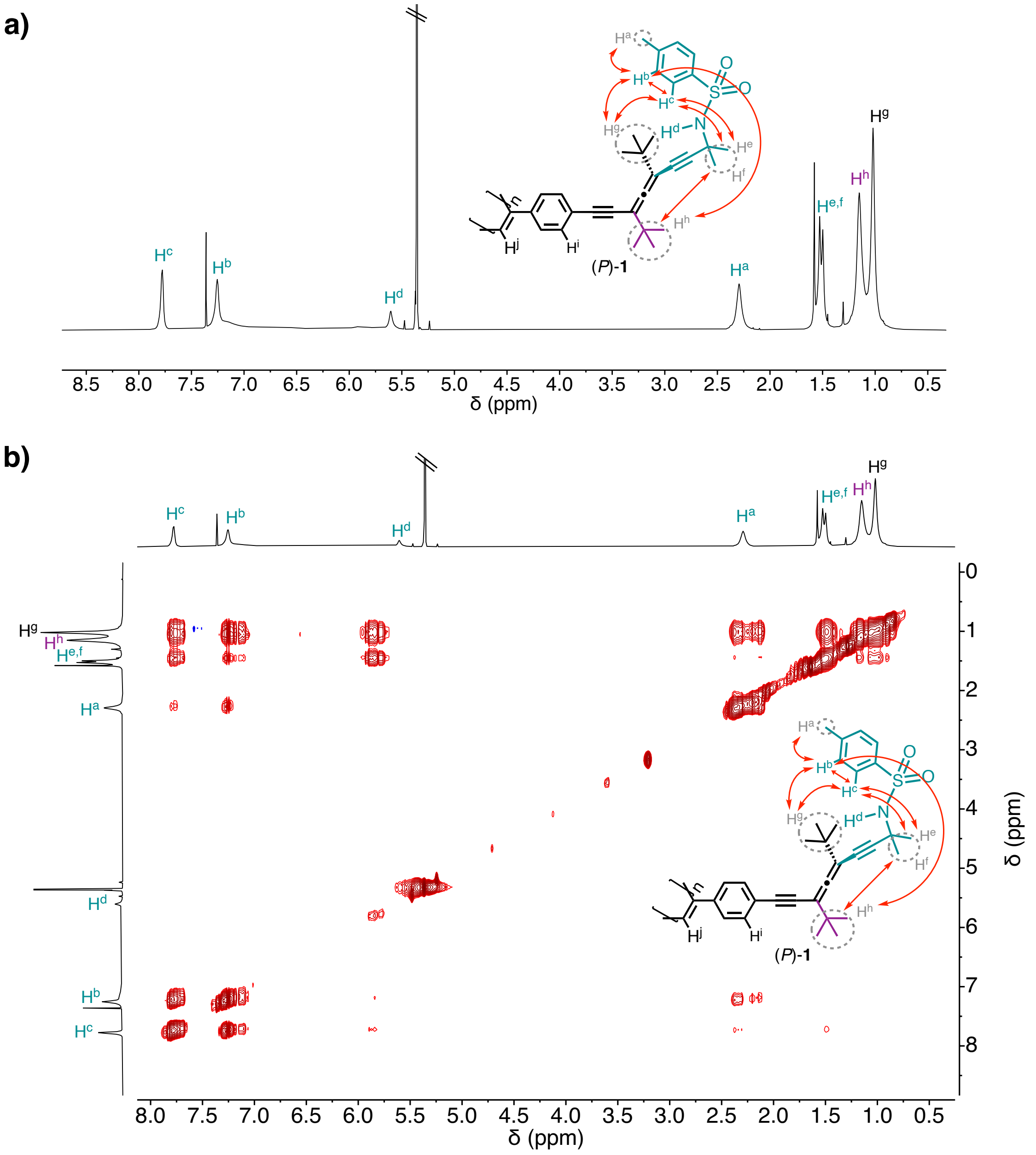

Figure S23. ^{1}H-NMR of poly-(*P*)-**1** (top) and NOESY (below) (CD_2Cl_2, 278 K, 750 MHz).

12. Low Temperature ECD Experiments

Poly-(*P*)-**1** (1.6 mM) in THF shows an increase in the ECD intensity after fast cooling (from 298 K to 268K at ≥10 K·min^{-1}). On the other hand, the ECD trace of poly-(*P*)-**1** (1.6 mM) in 1,2-DCE increases in intensity of the negative Cotton effect at 399 nm.

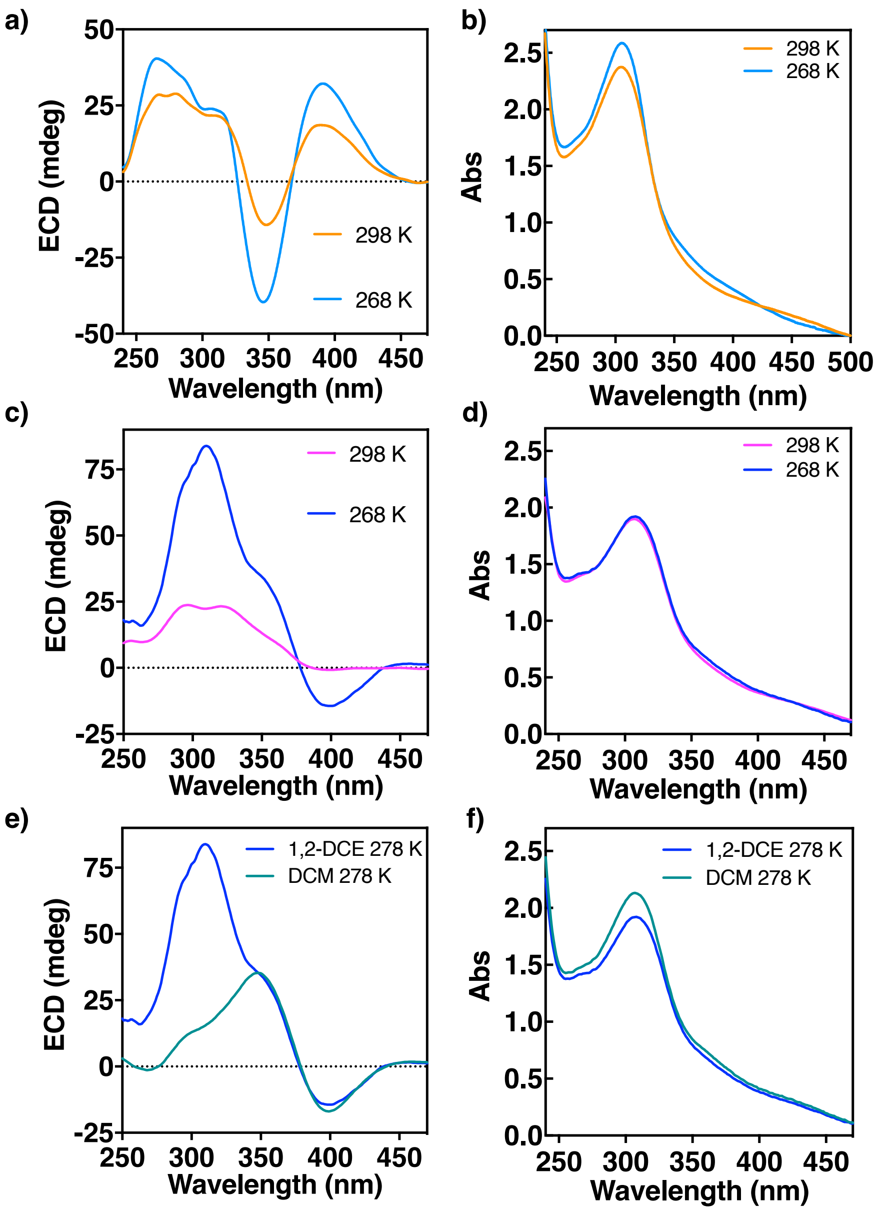

Figure S24. ECD spectra and UV-vis of poly-(*P*)-**1** (1.6 mM) at rt (298 K) and after fast cooling (at 268 K) in THF [(a), (b)] and in 1,2-DCE [(c), (d)]. ECD spectra (e) and UV-vis (f) of poly-(*P*)-**1** (1.6 mM) at 278 K in 1,2-DCE and DCM.

13. VT-ECD Experiments

*VT-ECD Experiments for poly-(P)-**1***

ECD signal of poly-(*P*)-**1** solutions (1.6 mM) were monitored during slow cooling from 298 K to 268 K at 0.1 K·min^{-1} (only spectra every 5 K were plotted for clarity).

Poly-(*P*)-**1** in THF shows an increase in the ECD intensity when the temperature decreases. On the other hand, the ECD trace of poly-(*P*)-**1** in 1,2-DCE increases in intensity

and takes place a bathochromic shift in the vinylic region from 400 nm to 420 nm with an increased intensity of that negative Cotton effect.

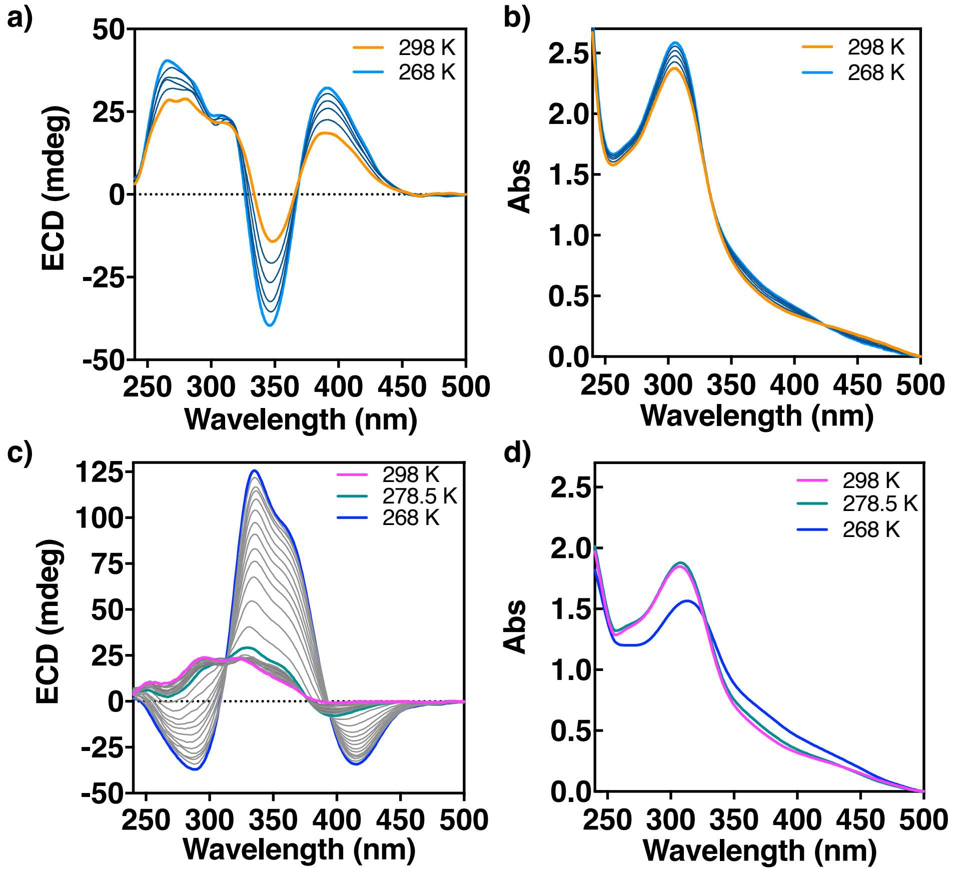

Figure S25. VT-ECD spectra and UV-vis of poly-(*P*)-**1** (1.6 mM) from 298 K to 268 K after slow cooling (0.1 K·min^{-1}) in THF [(a), (b)] and in 1,2-DCE [(c), (d)].

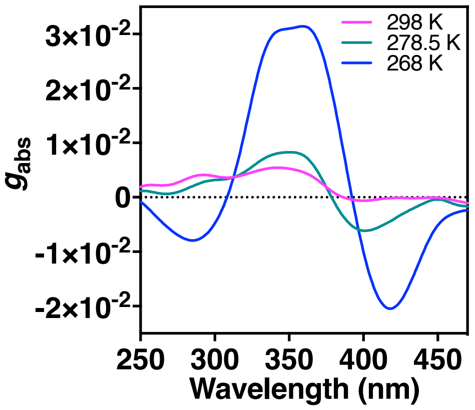

Figure S26. g_{abs} representation of poly-(*P*)-**1** (1.6 mM) from 298 K to 268 K after slow cooling (0.1 K·min^{-1}) in 1,2-DCE.

Also, several cycles of cooling-heating were carried out to corroborate the reversibility of the process (Figure S27).

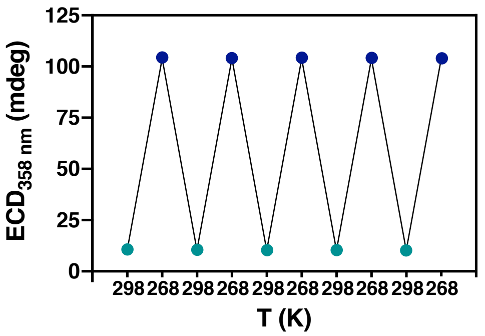

Figure S27. Temperature cycles by ECD at 358 nm.

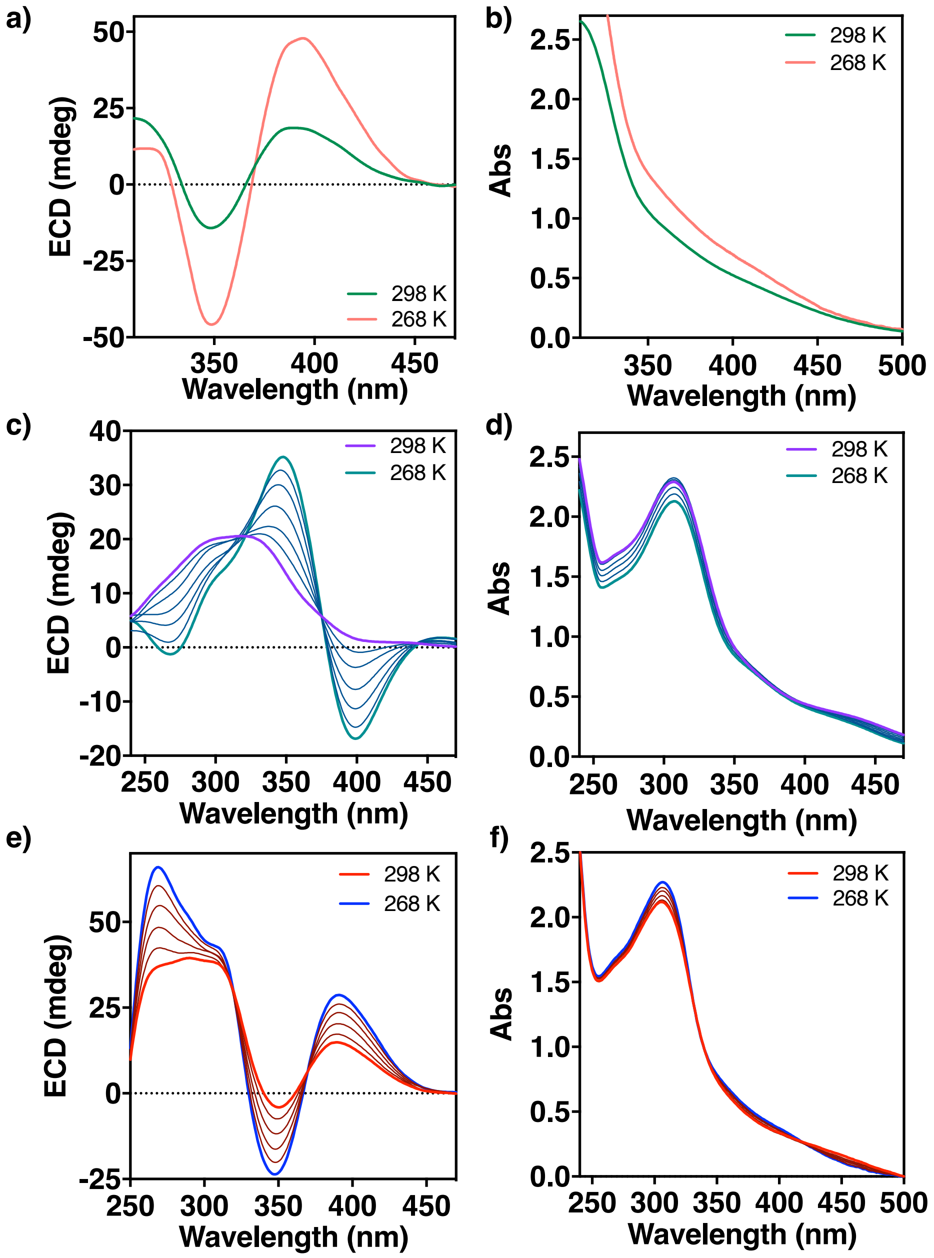

Figure S28. VT-ECD spectra and UV-vis of poly-(*P*)-**1** (1.6 mM) from 298 K to 268 K after slow cooling (0.1 K·min^{-1}) in DMF ((a), (b)), in DCM ((c), (d)) and in $CHCl_3$ ((e), (f)).

*VT-ECD Experiments for poly-(M)-**1***

VT-ECD experiments in THF and 1,2-DCE were repeated for the enantiomer poly-(*M*)-**1** and the same behaviour was observed.

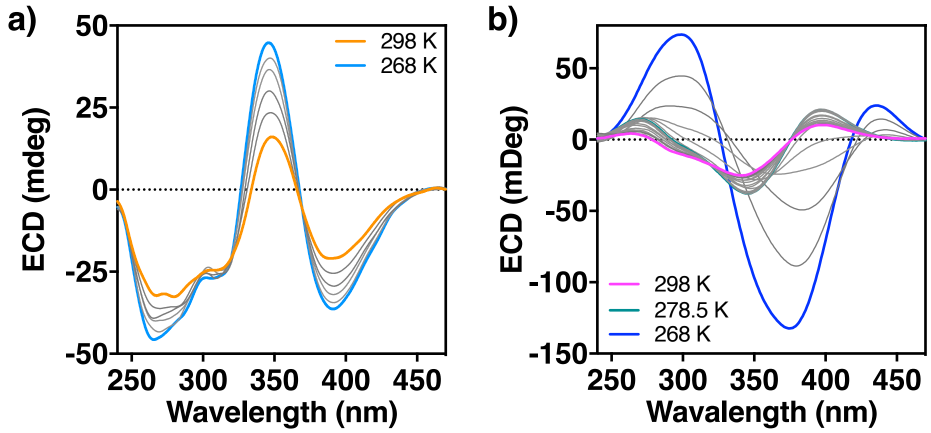

Figure S29. VT-ECD spectra of poly-(*M*)-**1** (1.6 mM) from 298 K to 268 K after slow cooling (0.1 K·min^{-1}) in THF (a) and in 1,2-DCE (c).

*VT-ECD Experiments for poly-(P)-**2***

ECD signal of poly-(*P*)-**2** solutions (0.8 mM) in Lewis base solvents (Figure S30) and non-Lewis base solvents (Figure S31) were monitored during fast cooling from 298 K to 268 K at 1.0 K·min^{-1} (only spectra every 5 K were plotted for clarity).

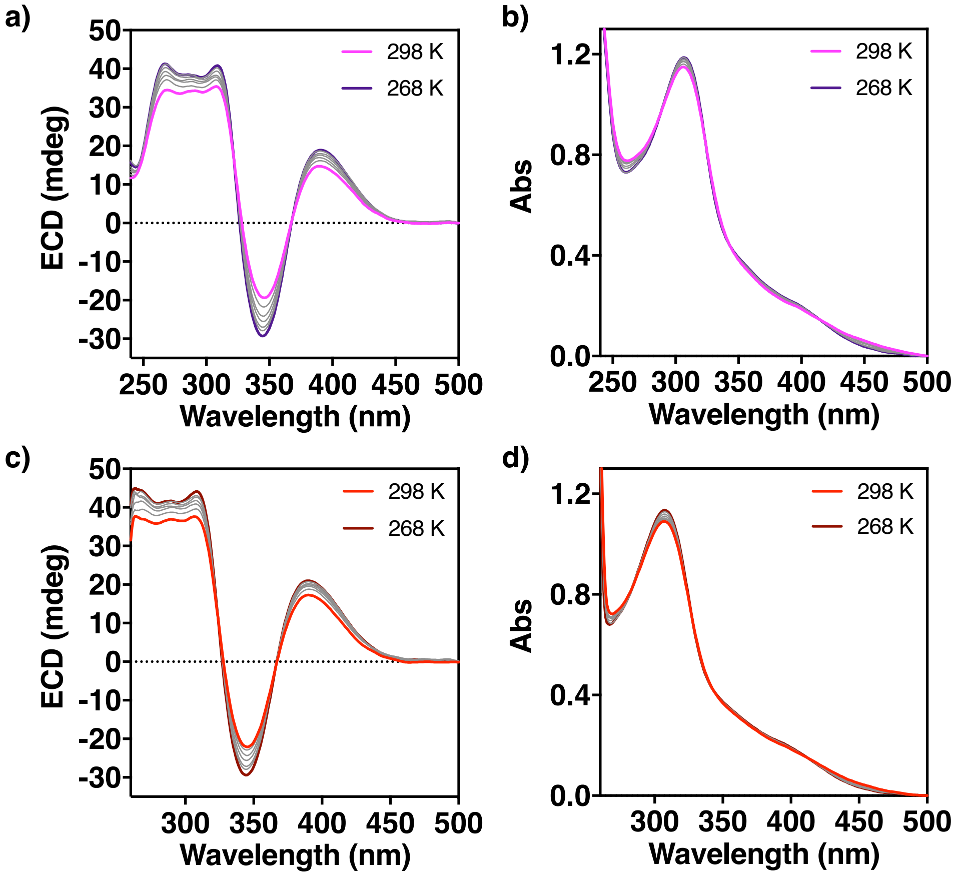

Figure S30. VT-ECD spectra and UV-vis of poly-(*P*)-**2** (0.8 mM) from 298 K to 268 K after fast cooling (1.0 K·min^{-1}) in THF ((a), (b)) and in DMF ((c), (d)).

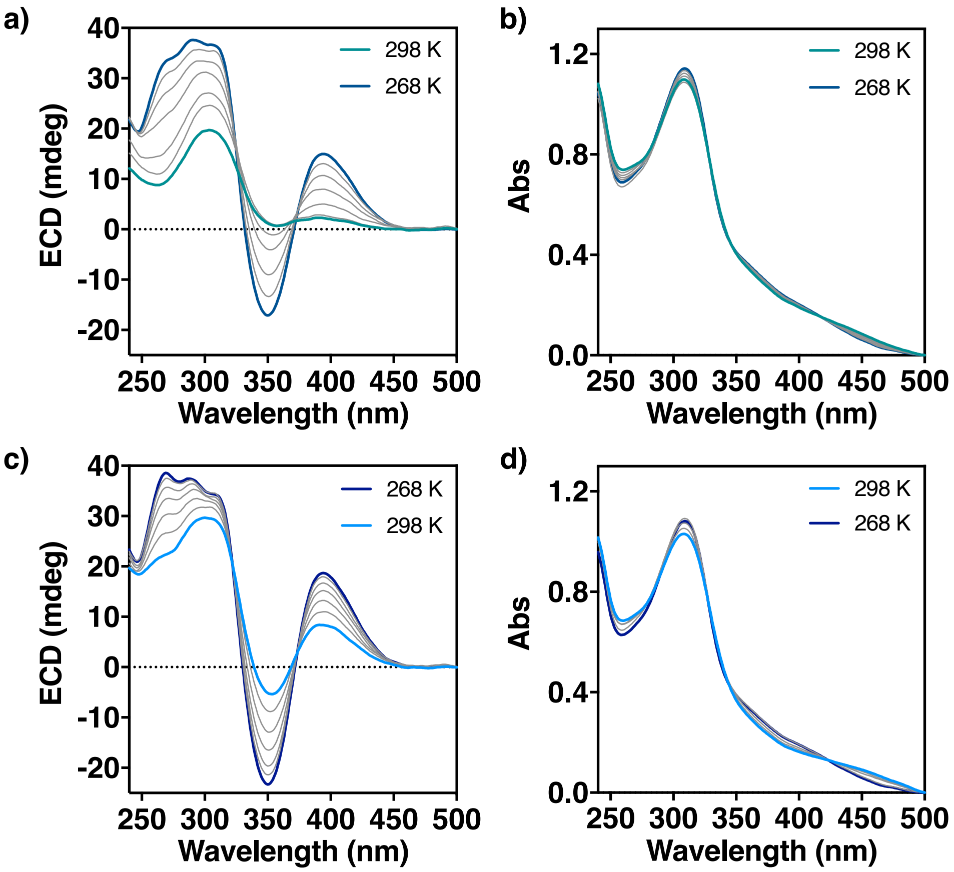

Figure S31. VT-ECD spectra and UV-vis of poly-(*P*)-**2** (0.8 mM) from 298 K to 268 K after fast cooling (1.0 K·min^{-1}) in 1,2-DCE ((a), (b)) and in DCM ((c), (d)).

ECD signal of poly-(*P*)-**2** solutions (0.8 mM) in Lewis base solvents (Figure S32) and non-Lewis base solvents (Figure S33) were monitored during slow cooling from 298 K to 268 K at 0.1 K·min^{-1} (only spectra every 2 K were plotted for clarity).

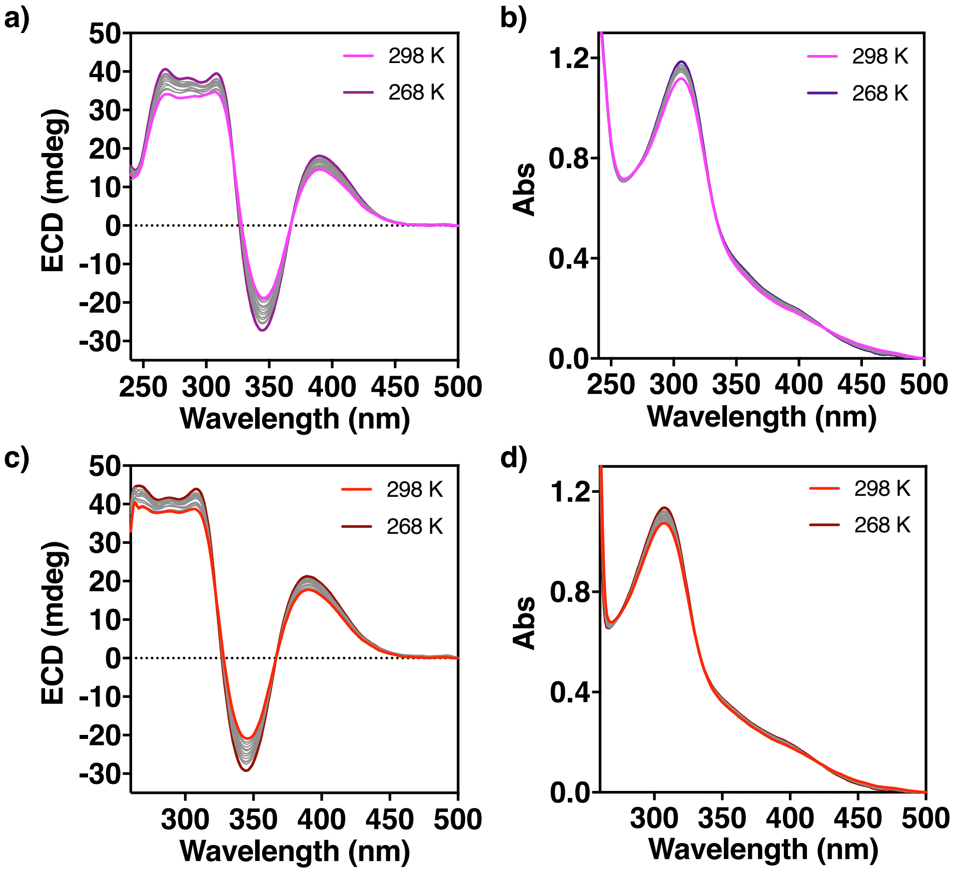

Figure S32. VT-ECD spectra and UV-vis of poly-(*P*)-**2** (0.8 mM) from 298 K to 268 K after fast cooling (1.0 K·min^{-1}) in THF ((a), (b)) and in DMF ((c), (d)).

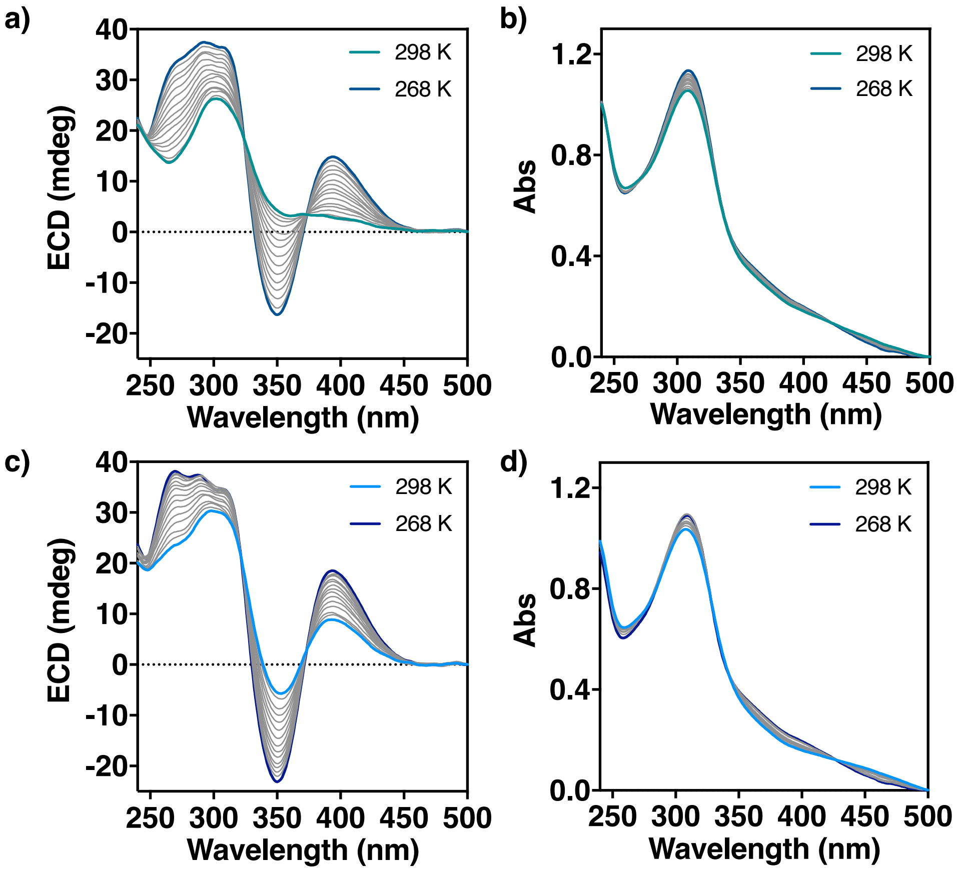

Figure S33. VT-ECD spectra and UV-vis of poly-(*P*)-**2** (0.8 mM) from 298 K to 268 K after fast cooling (1.0 K·min^{-1}) in 1,2-DCE ((a), (b)) and in DCM ((c), (d)).

14. Atomic Force Microscopy (AFM) Measurements for poly-(*P*)-1

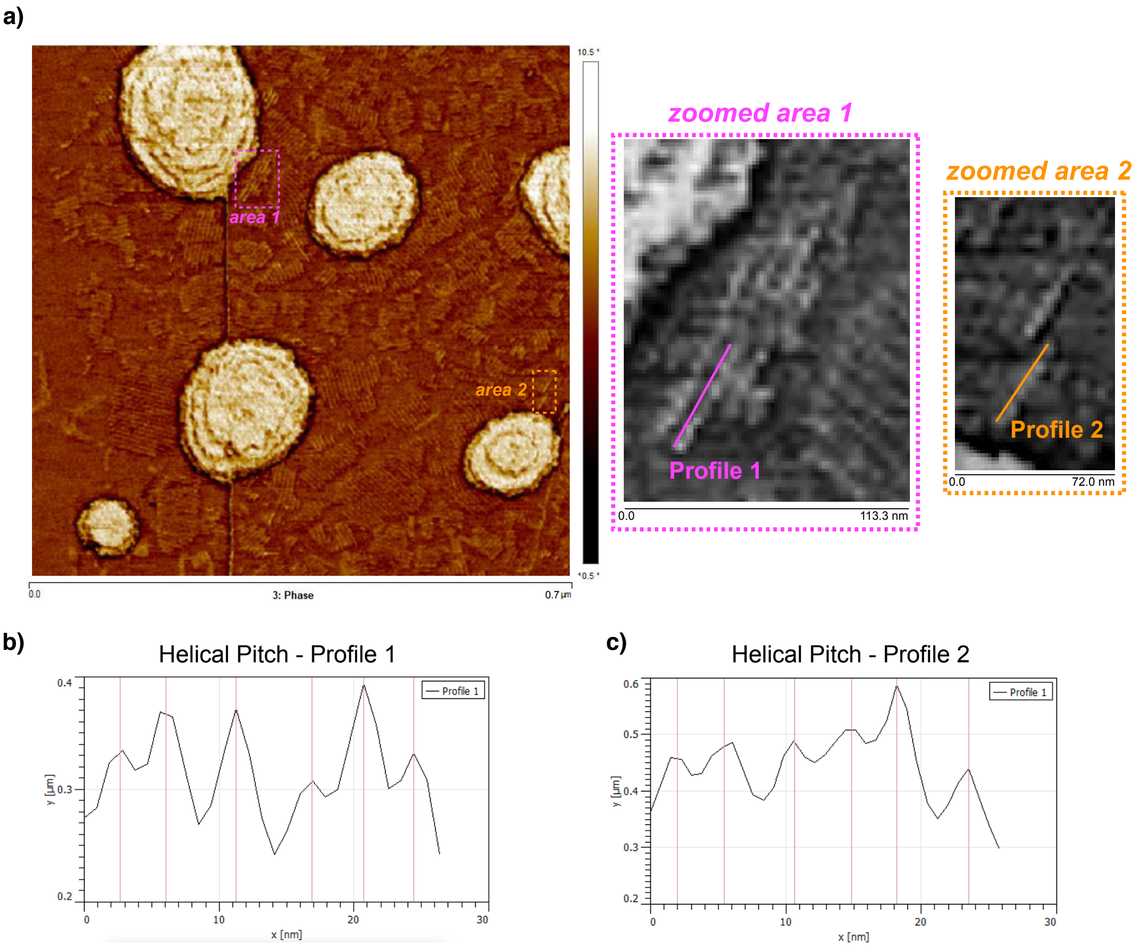

Puntos	X [nm]	Y [nm]	Longitud [nm]
	2.66	332.8	
	6.06	366.9	3.40
	11.26	370.6	5.20
	16.89	307.0	5.63
	20.72	392.8	3.84
	24.43	332.1	3.71

Puntos	X [nm]	Y [nm]	Longitud [nm]
	1.98	457.3	
	5.44	478.3	3.46
	10.64	487.8	5.20
	14.85	508.0	4.21
	18.19	588.3	3.34
	23.51	438.4	5.32

Figure S34. (a) AFM image for poly-(*P*)-**1** (prepared from a THF solution) and zoomed areas or the highlighted regions. (b, c) Graphics depicting the helical pitch profile measured in the indicated areas.

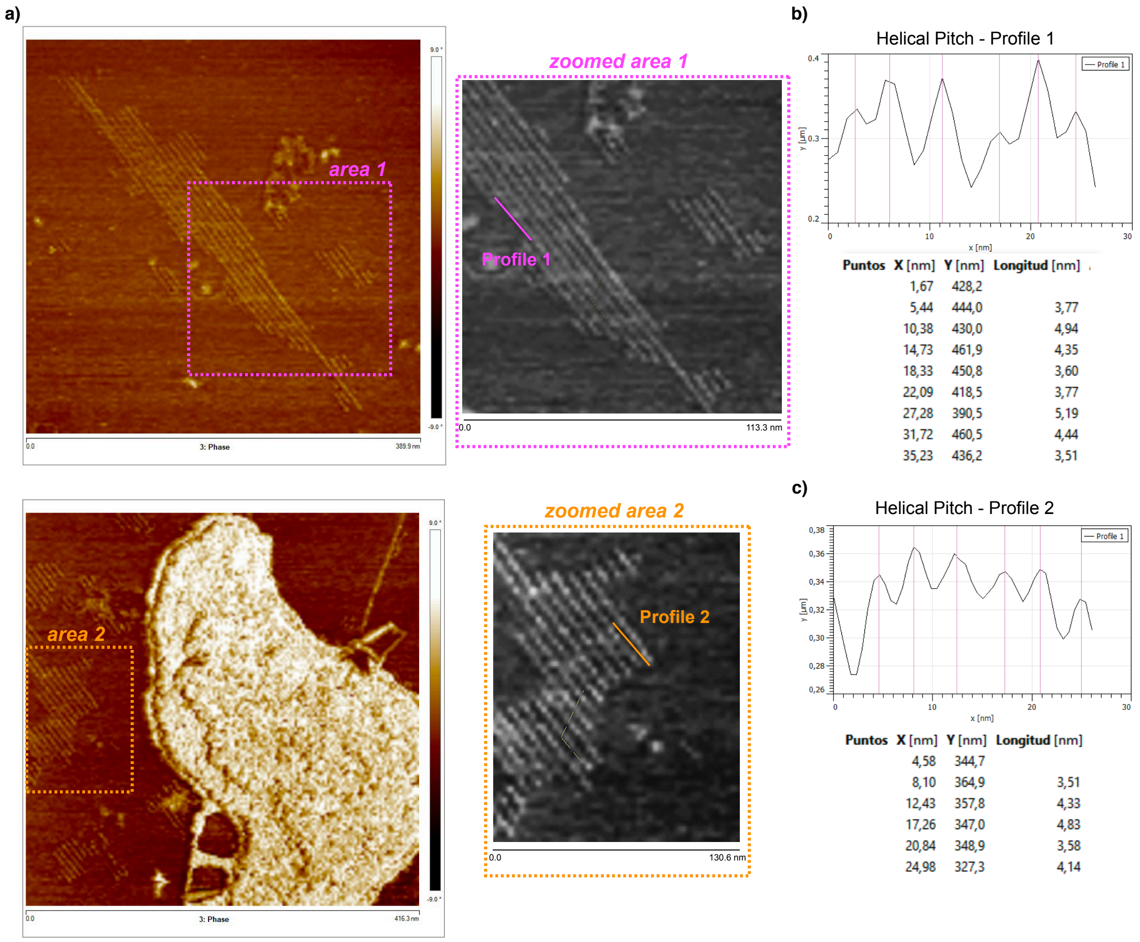

Figure S35. (a) AFM image for poly-(*P*)-**1** (prepared from a 1,2-DCE solution) and zoomed areas or the highlighted regions. (b, c) Graphics depicting the helical pitch profile measured in the indicated areas.

15. Theoretical Calculations

Considering the difficulties to carry out ECD theoretical calculations on large polymers, representative oligomers were used. So, an oligomer of poly-(*P*)-**1** with n= 9 —where n denotes the number of monomer repeating units (mru)— was employed. The number of monomer units was selected considering the results of previous studies,[S4-S6] where our group evaluated the spectra for a series of poly(phenylacetylene) (PPA) oligomers obtained through systematic increase of monomer units, and concluded that 8-10 monomers were enough to describe the n+2 polymer ECD spectra. The starting structure of poly-(*P*)-**1** was built through adjustment to the experimental data obtained from structural techniques, such as AFM, DSC and UV-vis spectroscopy, defining the four different dihedral angles needed to build up the helical scaffold (ω_1, ω_2, ω_3 and ω_4; see

Figure S36). Additionally, the pendant groups were introduced in the most stable conformation.

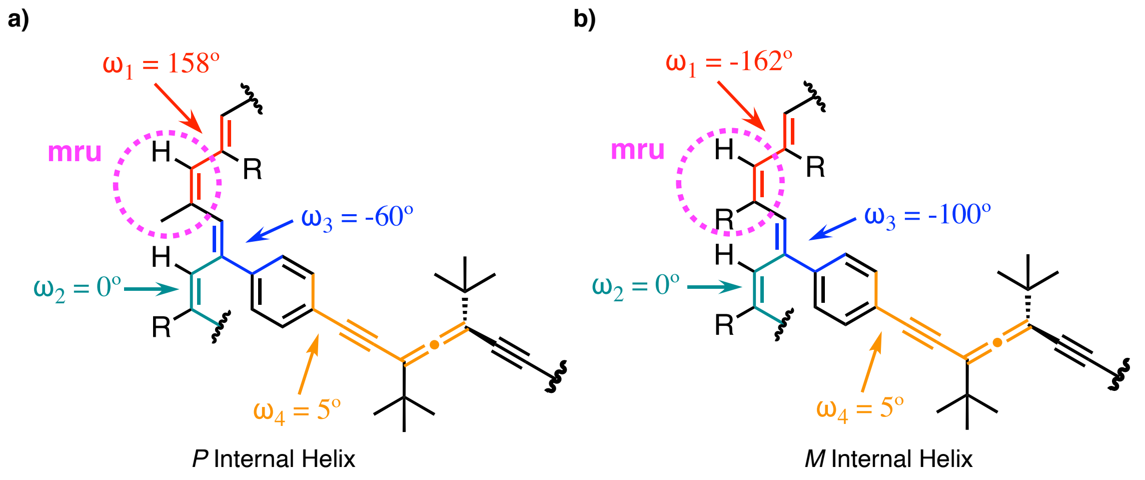

Figure S36. Main dihedral angles involved in the helical structure of PAEPAs and derivatives and the corresponding values obtained for the *P* Helix (a) and for the *M* Helix (b).

The ECD computational methodology was selected according to the size of polymers under investigation. Taking this into account, to evaluate the theoretical spectra time dependent density functional theory (TD-DFT),[S7] in combination with the CAM-B3LYP functional[S8] and the 3-21G basis set,[S9] have been used (including 80 excitations). All computations were carried out using Gaussian-16 (G16RevC.01).[S10]

The full width at half height (FWHM) was fixed to 20.0 nm and the ECD were plotted with Gaussian curves. For an efficient comparison and considering the tendency of the TD-DFT method to overestimate the excitation energies, the wavelength and intensity at the maximum/minimum Cotton effect correspondent to the polyene backbone in the theoretical spectra were adjusted to the experimental spectra. Employing the same correction factors, the lambdas were shifted, and the intensities rescaled. The resulting ECD spectra is in good agreement with the experimental ones (Figure S37, S38).

a)

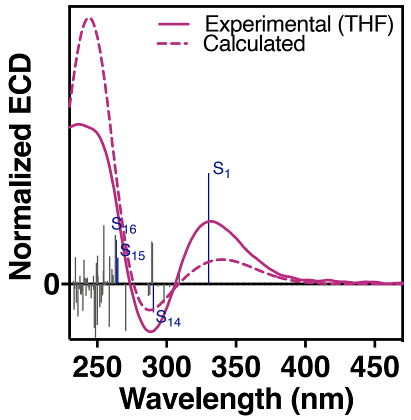

b)

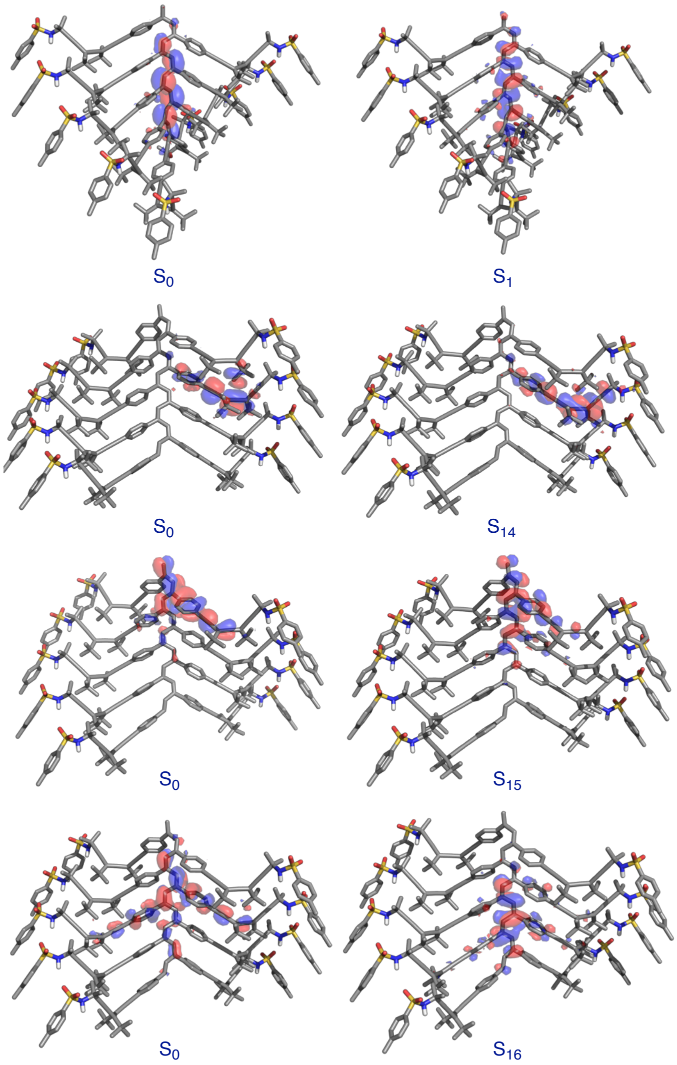

Figure S37. (a) TD-DFT (CAM-B3LYP)/3-21G ECD spectrum for poly-(*P*)-**1**, showing the excited states that contribute the most to the Cotton bands, vs. ECD experimental spectra of poly-(*P*)- in THF. (b) Electron density differences with respect to the ground state for S0 to S1, S0 to S14, S0 to S15 and S0 to S16.

a)

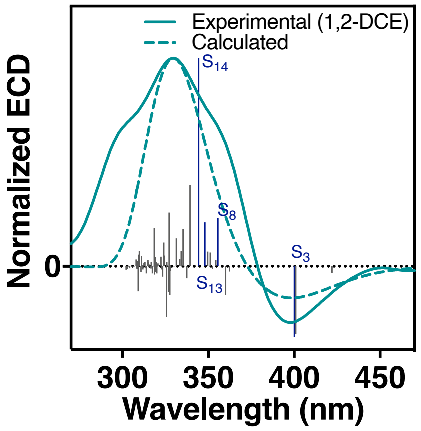

b)

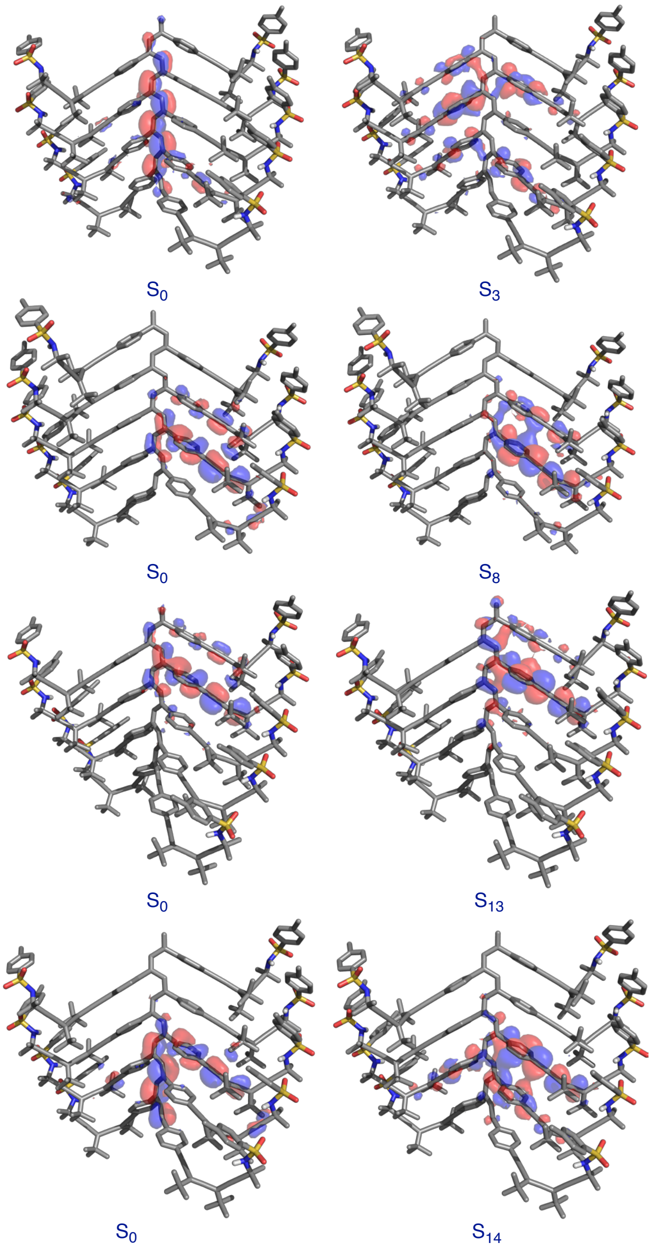

Figure S38. (a) TD-DFT (CAM-B3LYP)/3-21G ECD spectrum for poly-(*P*)-**1**, showing the excited states that contribute the most to the Cotton bands, vs. ECD experimental spectra of poly-(*P*)-**1**

in 1,2-DCE. (b) Electron density differences with respect to the ground state for S0 to S1, S0 to S8, S0 to S13 and S0 to S14.

16. ECD and UV-vis studies of poly-(*P*)-1 in presence of different anions

Solutions of poly-(*P*)-**1** 0.8 mM in anhydrous THF and 1.60 mM in anhydrous 1,2-DCE of the corresponding monomers were measured in the presence of tetrabutylammonium (TBA) salts derived from N_3^-, CN^-, F^- anions. TBA salts were dried for 12 h at 323 K under high vacuum. Then, these salts were dissolved in anhydrous MeCN.

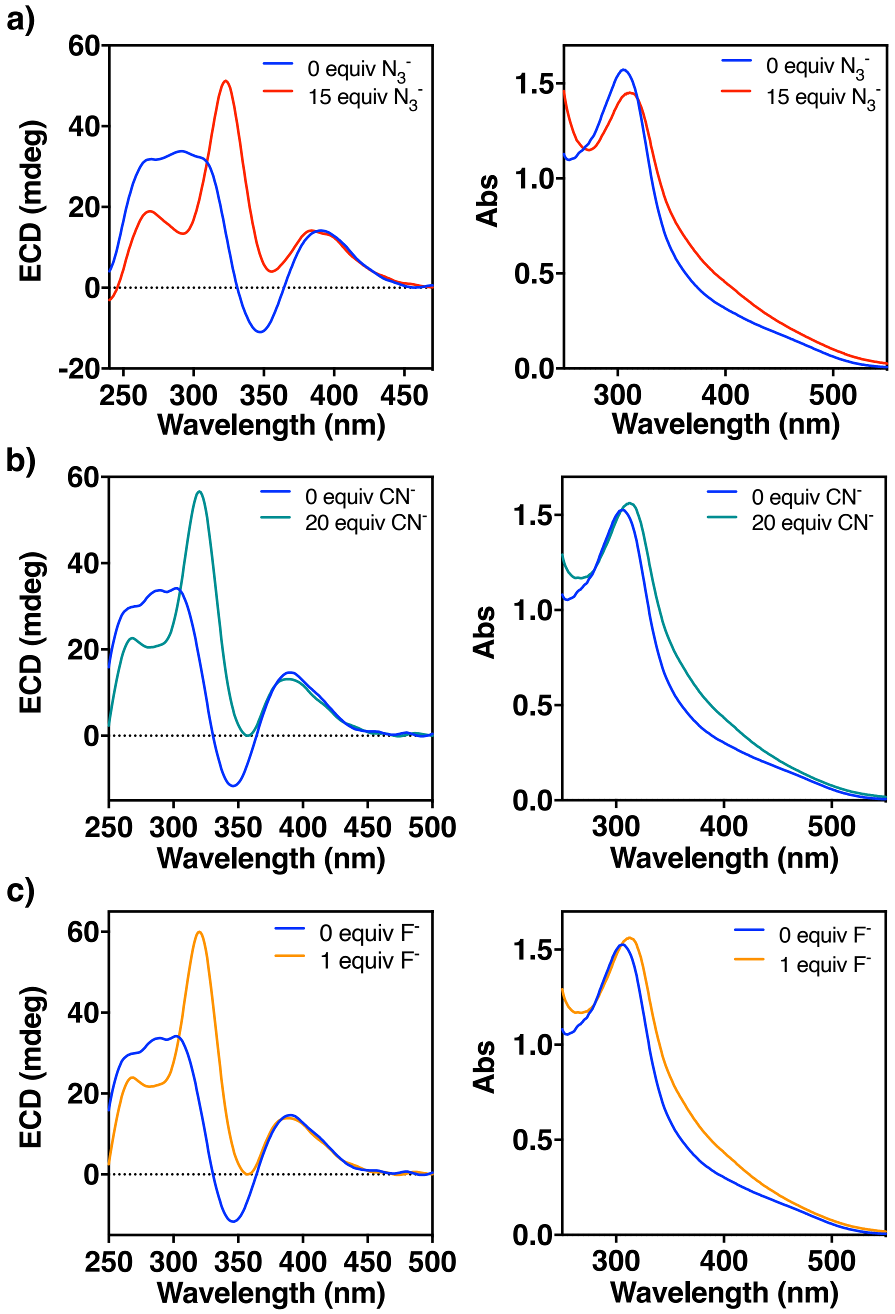

Figure S39. ECD and UV-vis spectra of poly-(*P*)-**1** in THF (0.80 mM) titrated with different amounts of anions (0.35 mM in MeCN) at rt (298 K).

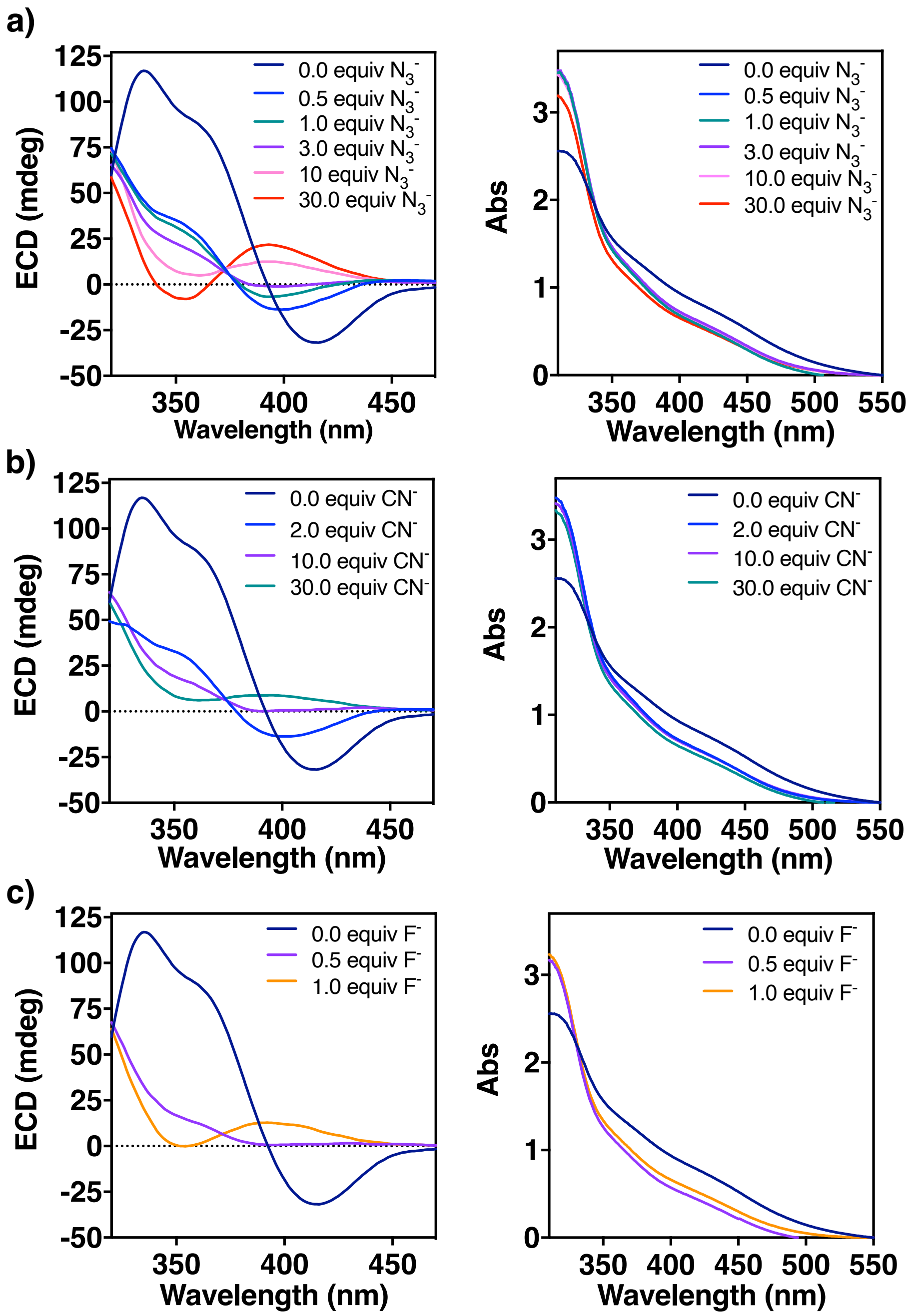

Figure S40. ECD and UV-vis spectra of poly-(*P*)-**1** in 1,2-DCE (1.60 mM) titrated with different amounts of anions (0.35 mM in MeCN) at 268 K (cooling at 0.1 K·min^{-1}).

Furthermore, the reversibility of the process was demonstrated by washing the organic solution with aqueous media to remove the anions (2 x 2mL). The resulting organic phase was dried over anhydrous Na_2SO_4 and after evaporation *in vacuo*, the sample was redissolved in 1,2-DCE (1.6 mM), recovering the original helical sense as shown by ECD (Figure S41).

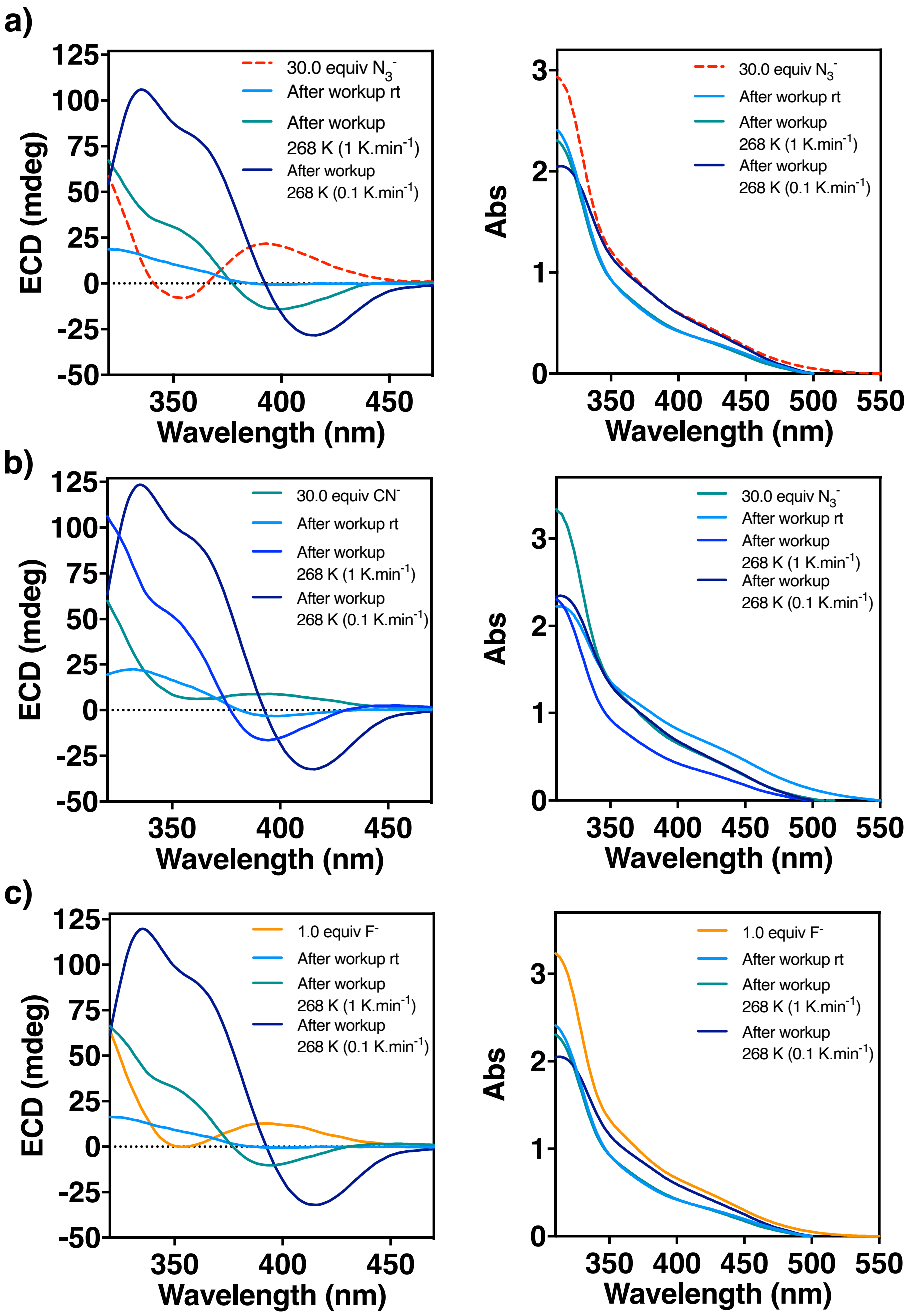

Figure S41. ECD and UV-vis spectra of poly-(*P*)-**1** in 1,2-DCE (1.60 mM) titrated with different amounts of anions (0.35 mM in MeCN) at 268 K (cooling at 0.1 K·min^{-1}) and the data after anions removal.

17. NMR Anion Titration Experiments

^{1}H NMR experiments were measure to solutions of mono-(*P*)-**1** and poly-(*P*)-**1** (5.9 mM) in THF-d_8 and in CD_2Cl_2 upon addition of tetrabutylammonium (TBA) salts derived from N_3^-, F^- anions (0.38 mM in MeCN). TBA salts were dried for 12 h at 323 K under high vacuum. Then, these salts were dissolved in anhydrous MeCN.

*NMR studies of the mono-(P)-**1** in the presence of different anions*

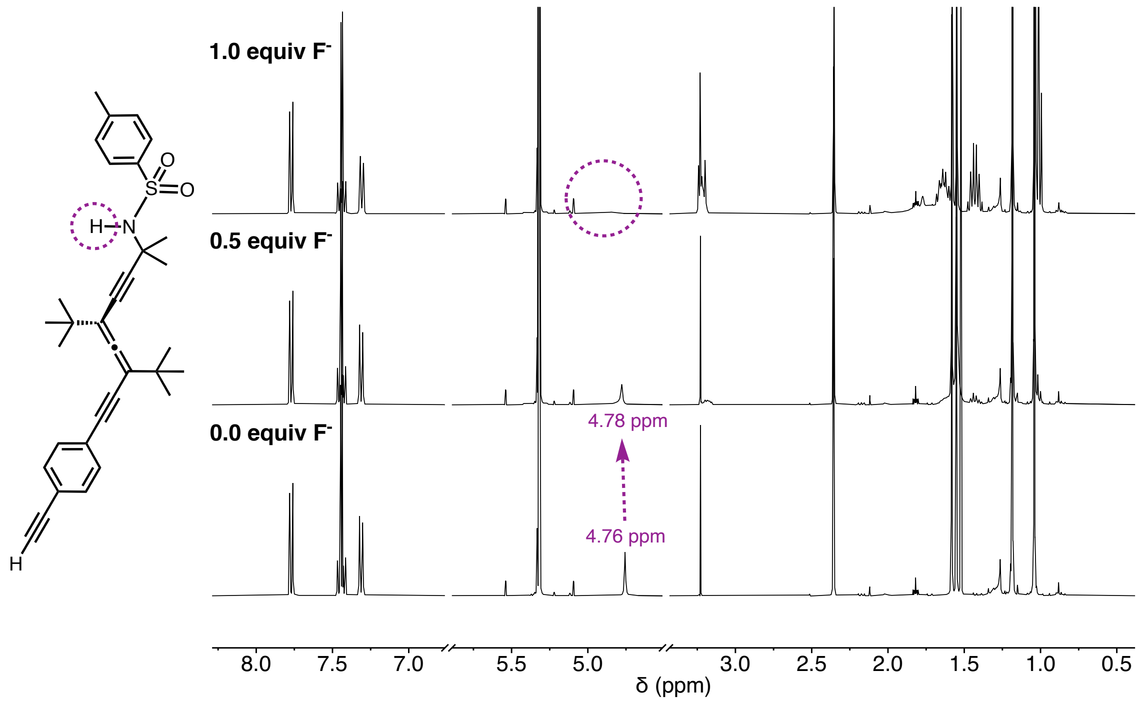

Figure S42. ^{1}H-NMR of mono-(*P*)-**1** in the presence of different amounts of TBAF (300 MHz, CD_2Cl_2).

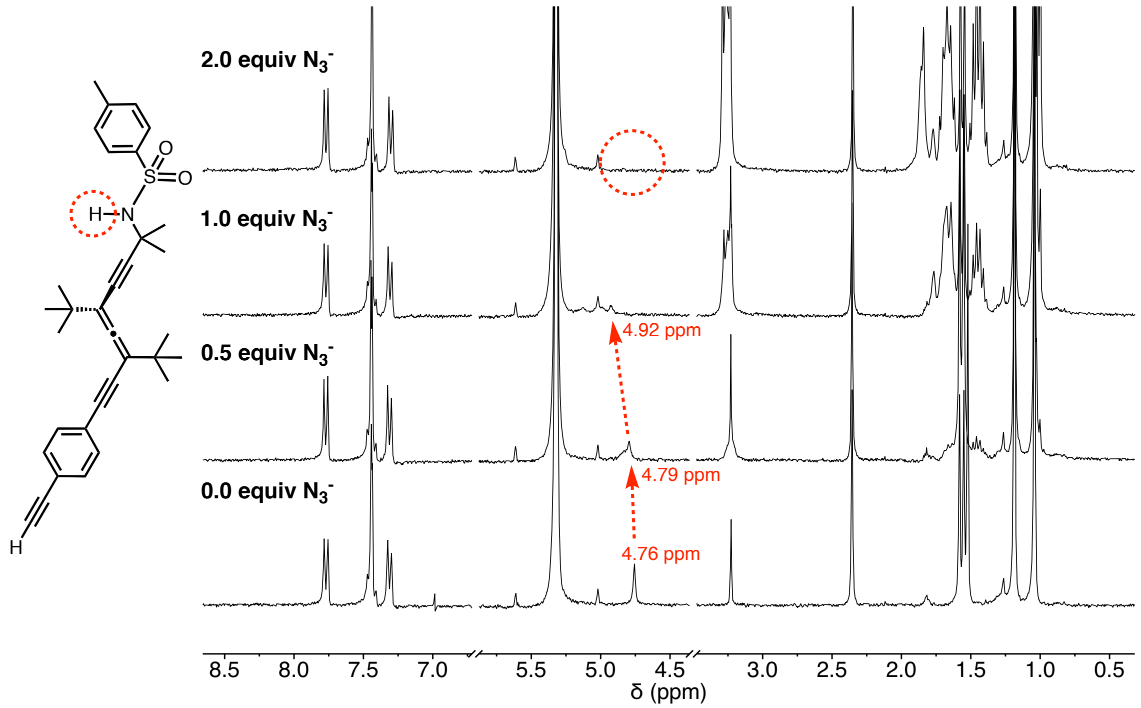

Figure S43. ^{1}H-NMR of mono-(*P*)-**1** in the presence of different amounts of $TBAN_3$ (300 MHz, CD_2Cl_2).

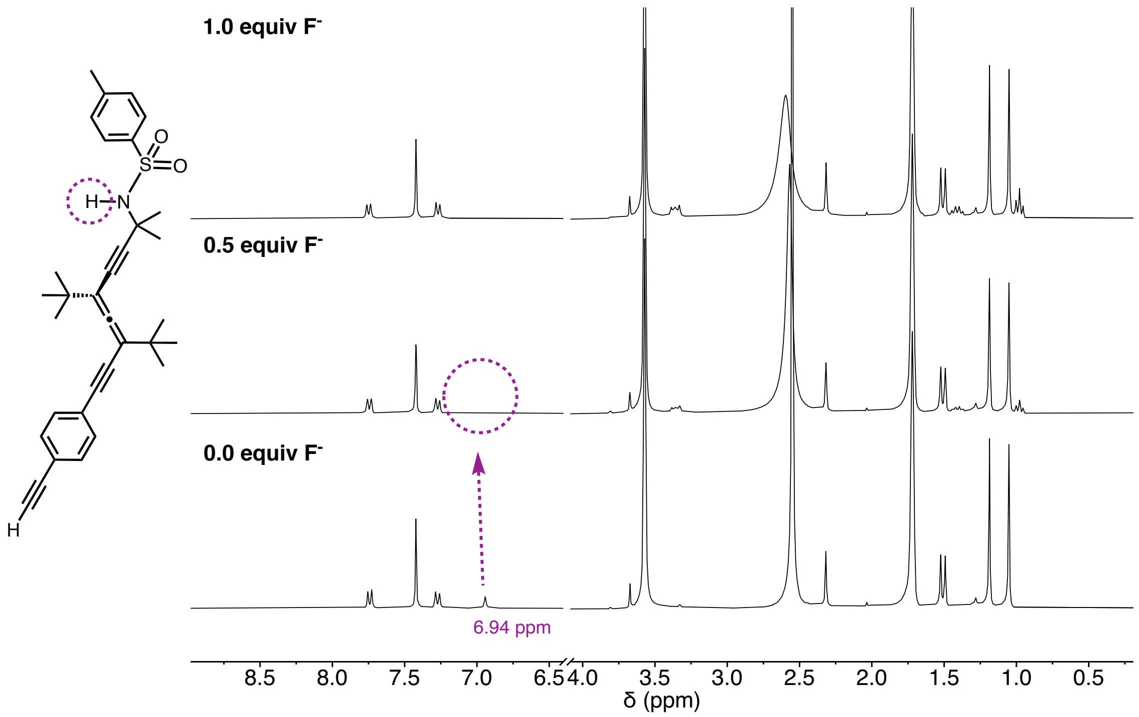

Figure S44. ^{1}H-NMR of mono-(*P*)-**1** in the presence of different amounts of TBAF (300 MHz, THF-d_8).

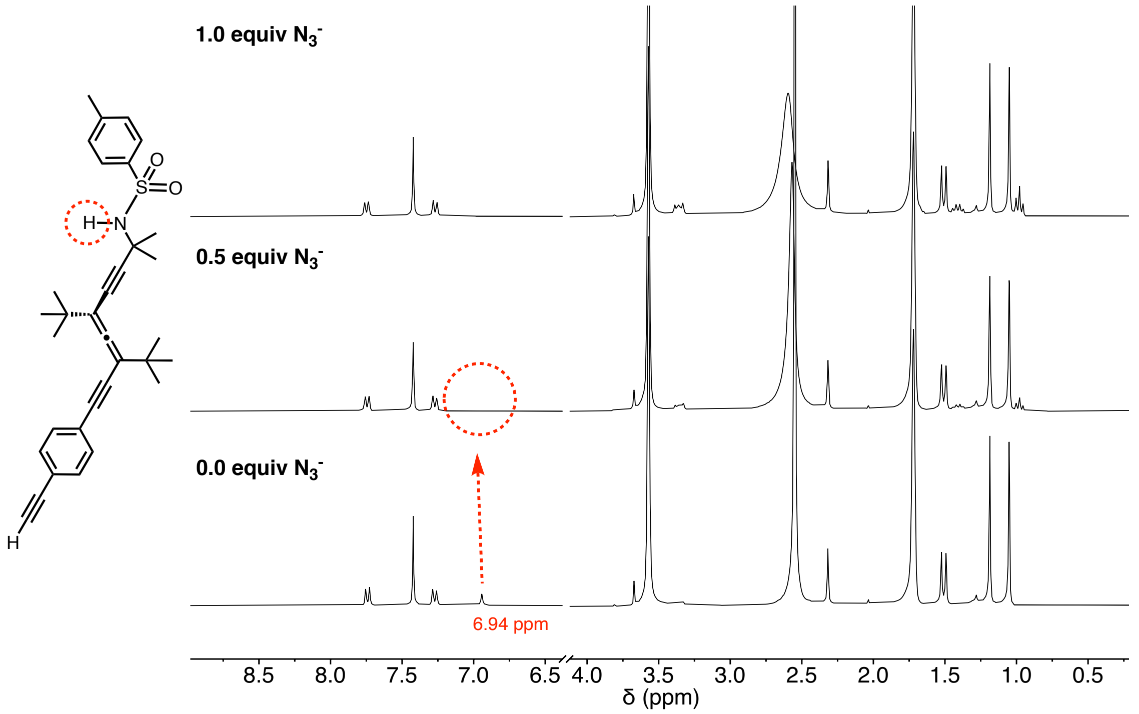

Figure S45. ^{1}H-NMR of mono-(*P*)-**1** in the presence of different amounts of $TBAN_3$ (300 MHz, THF-d_8).

*NMR studies of the poly-(P)-**1** in the presence of different anions*

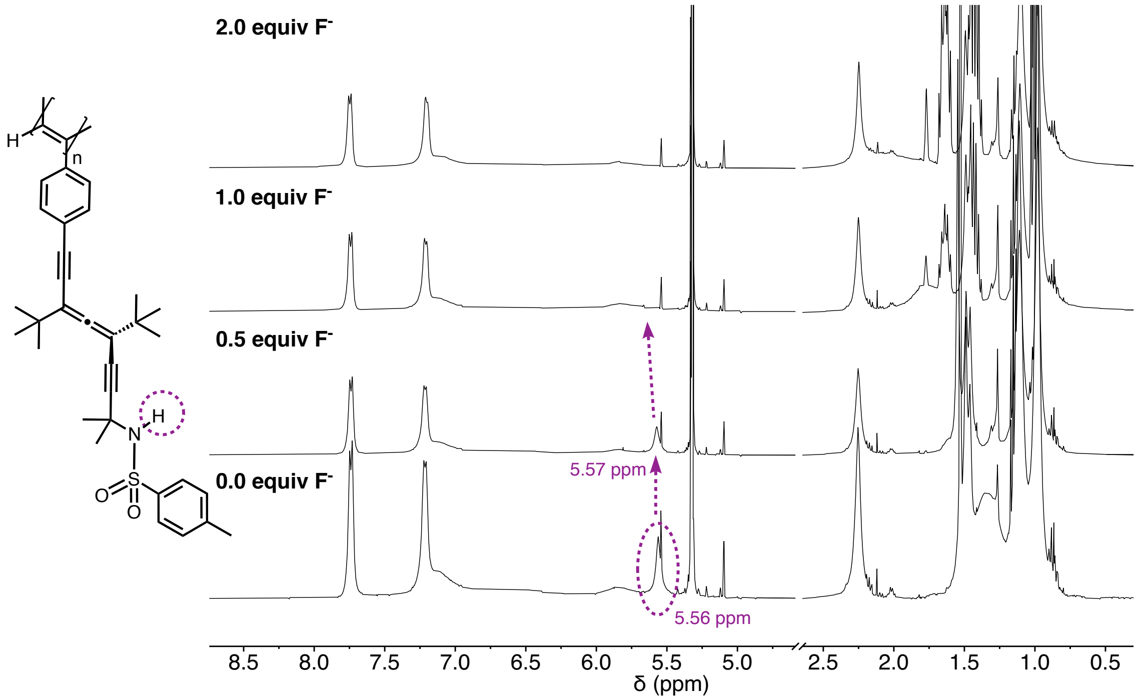

Figure S46. ^{1}H-NMR of poly-(*P*)-**1** in the presence of different amounts of TBAF (300 MHz, CD_2Cl_2).

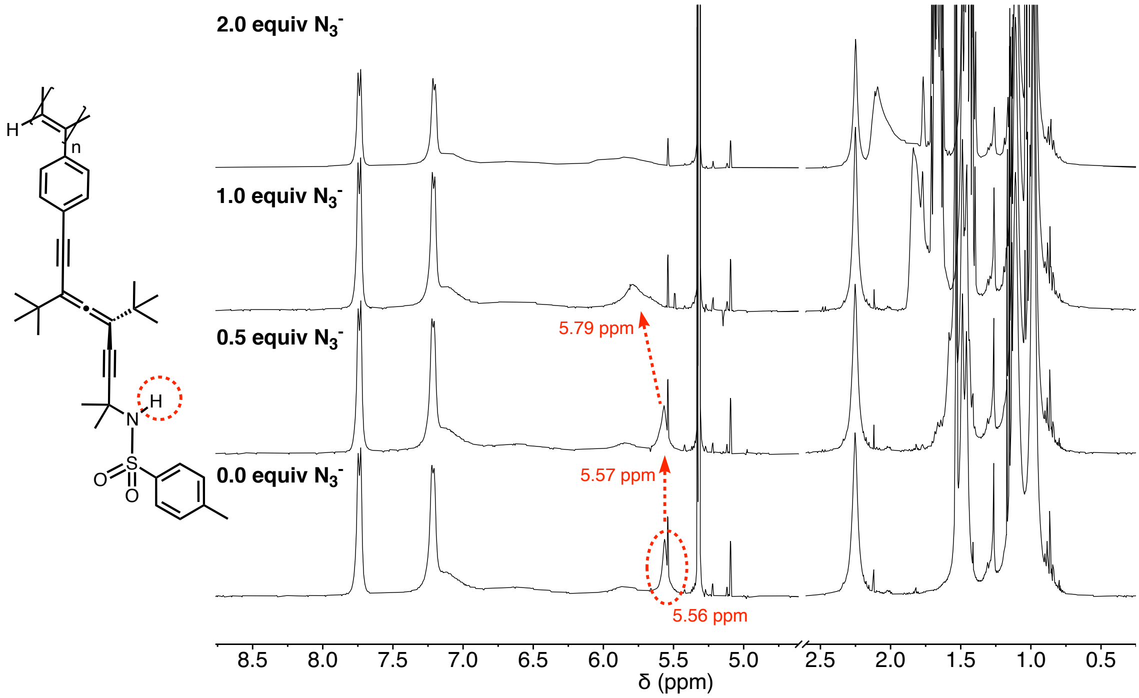

Figure S47. ^{1}H-NMR of poly-(*P*)-**1** in the presence of different amounts of $TBAN_3$ (300 MHz, CD_2Cl_2).

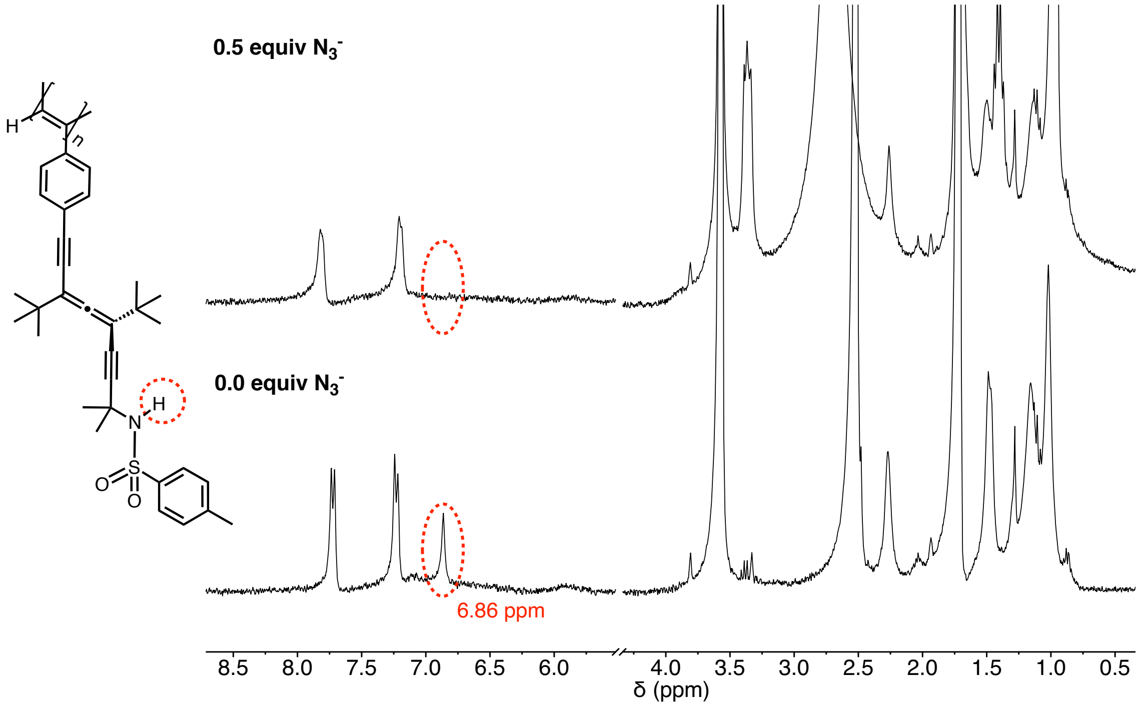

Figure S48. ^{1}H-NMR of poly-(*P*)-**1** in the presence of different amounts of $TBAN_3$ (300 MHz, THF-d_8).

18. References

(S1) Lago-Silva, M.; Cid, M. M.; Quiñoá, E.; Freire, F. Dynamic Axial-to-Helical Communication Mechanism in Poly[(allenylethynylenephenylene)acetylene]s under External Stimuli. *Angew. Chem. Int. Ed.* **2023**, *62*, e202303329.
(S2) Liu, L.; Namikoshi, T.; Zang, Y.; Aoki, Y.; Hadano, S.; Abe, Y.; Wasuzu, I.; Tsutsuba, T.; Teraguchi, M.; Kaneko, T. J. Top-Down Preparation of Self-Supporting Supramolecular Polymeric Membranes Using Highly Selective Photocyclic Aromatization of Cis–Cisoid Helical Poly(phenylacetylene)s in the Membrane Stat. *J. Am. Chem. Soc.* **2013**, *135*, 602.
(S3) Leiras, S.; Freire, F.; Seco, J. M.; Quiñoá, E.; Riguera, R. Controlled modulation of the helical sense and the elongation of poly(phenylacetylene)s by polar and donor effects. *Chem. Sci.* **2013**, *4*, 2735-2743.
(S4) Fernández, B.; Rodríguez, R.; Rizzo, A.; Quiñoá, E.; Riguera, R.; Freire, F. Predicting the Helical Sense of Poly(phenylacetylene)s from their Electron Circular Dichroism Spectra. *Angew. Chem. Int. Ed.* **2018**, *57*, 3666-3670.
(S5) Fernández, B.; Rodríguez, R.; Quiñoá, E.; Riguera, R.; Freire, F. Decoding the ECD Spectra of Poly(phenylacetylene)s: Structural Significance. *ACS Omega* **2019**, *4*, 5233-5240.
(S6) Fernández, Z.; Fernández, B.; Quiñoá, E.; Riguera, R.; Freire, F. Chiral information harvesting in helical poly(acetylene) derivatives using oligo(p-phenyleneethynylene)s as spacers. *Chem. Sci.* **2020**, *11*, 7182-7187.
(S7) Runge, E.; Gross, E. K. U. Density-Functional Theory for Time-Dependent Systems *Phys. Rev. Lett.* **1984**, *52*, 997-1000.
(S8) Yanai, Y.; Tew, D. P.; Handy, N. C. A. A new hybrid exchange–correlation functional using the Coulomb-attenuating method (CAM-B3LYP). *Chem. Phys. Lett.* **2005**, *393*, 51-57.
(S9) Binkley, J. S.; Pople, J. A.; Hehre, W. J. Self-Consistent Molecular Orbital Methods. 21. Small Split-Valence Basis Sets for First-Row Elements. *J. Am. Chem. Soc.* **1980**, *102*, 939-947.
(S10) Frisch, M. J.; Trucks, G. W.; Schlegel, H. B.; Scuseria, G. E.; Robb, M. A.; Cheeseman, J. R.; Scalmani, G.; Barone, V.; Petersson, G. A. ; Nakatsuji, H.; Li, X.; Caricato, M.; Marenich, A. V.;

Bloino, J.; Janesko, B. G.; Gomperts, R.; Mennucci, B.; Hratchian, H. P.; Ortiz, J. V.; Izmaylov, A. F.; Sonnenberg, J. L.; Williams-Young, D.; Ding, F.; Lipparini, F.; Egidi, F.; Goings, J.; Peng, B.; Petrone, A.; Henderson, T.; Ranasinghe, D.; Zakrzewski, V. G.; Gao, J.; Rega, N.; Zheng, G.; Liang, W.; Hada, M.; Ehara, M.; Toyota, K.; Fukuda, R.; Hasegawa, J.; Ishida, M.; Nakajima, T.; Honda, Y.; Kitao, O.; Nakai, H.; Vreven, T.; Throssell, K. ; Montgomery, J. A.; Peralta, Jr., J. E. ; Ogliaro, F.; Bearpark, M. J.; Heyd, J. J.; Brothers, E. N. ; Kudin, K. N.; Staroverov, V. N. ; Keith, T. A. ; Kobayashi, R.; Normand, J.; Raghavachari, K.; Rendell, A. P.; Burant, J. C.; Iyengar, S. S.; Tomasi, J.; Cossi, M.; Millam, J. M. ; Klene, M.; Adamo, C.; Cammi, R.; Ochterski, J. W.; Martin, R. L.; Morokuma, K.; Farkas, O.; Foresman, J. B.; Fox, D. J. Gaussian, Inc. Gaussian 16, Revision C.01. Wallingford, CT: Gaussian, Inc.; 2016.
